# Passive transport of Ca^2+^ ions through lipid bilayers imaged by widefield second harmonic microscopy

**DOI:** 10.1016/j.bpj.2023.01.018

**Published:** 2023-01-19

**Authors:** Maksim Eremchev, David Roesel, Chetan S. Poojari, Aurélien Roux, Jochen S. Hub, Sylvie Roke

**Affiliations:** 1 Laboratory for Fundamental BioPhotonics (LBP), Institute of Bioengineering (IBI), School of Engineering (STI), École Polytechnique Fédérale de Lausanne (EPFL), Lausanne, Switzerland; 2 Theoretical Physics and Center for Biophysics, Saarland University, Saarbrücken, Germany; 3 Biochemistry Department, University of Geneva, Geneva, Switzerland; 4 Swiss National Centre for Competence in Research Programme Chemical Biology, Geneva, Switzerland; 5 School of Chemistry and Biochemistry, University of Geneva, Geneva, Switzerland; 6 Institute of Materials Science and Engineering (IMX), School of Engineering (STI), École Polytechnique Fédérale de Lausanne (EPFL), Lausanne, Switzerland; 7 Lausanne Centre for Ultrafast Science, École Polytechnique Fédérale de Lausanne (EPFL), Lausanne, Switzerland

## Abstract

In biology, release of Ca^2+^ ions in the cytosol is essential to trigger or control many cell functions. Calcium signaling acutely depends on lipid membrane permeability to Ca^2+^. For proper understanding of membrane permeability to Ca^2+^, both membrane hydration and the structure of the hydrophobic core must be taken into account. Here, we vary the hydrophobic core of bilayer membranes and observe different types of behavior in high-throughput wide-field second harmonic imaging. Ca^2+^ translocation is observed through mono-unsaturated (DOPC:DOPA) membranes, reduced upon the addition of cholesterol, and completely inhibited for branched (DPhPC:DPhPA) and poly-unsaturated (SLPC:SLPA) lipid membranes. We propose, using molecular dynamics simulations, that ion transport occurs through ion-induced transient pores, which requires nonequilibrium membrane restructuring. This results in different rates at different locations and suggests that the hydrophobic structure of lipids plays a much more sophisticated regulating role than previously thought.

## Significance

Interaction of Ca^2+^ ions with lipid membranes regulates a variety of processes like membrane fusion, fission, and signaling. It is generally accepted that Ca^2+^ ions do not passively permeate through pure lipid membranes of any kind. However, the impermeability to Ca^2+^ ions is not compatible with recently proposed fusion and fission mechanisms, which logically predict that Ca^2+^ ions should be able to translocate through membranes (albeit at low rates). Here, we revisit the question of Ca^2+^ ion translocation through lipid membranes, by performing molecular interface specific measurements complemented by molecular dynamics simulations and standard fluorescence measurements and find that passive ion permeation does occur. Our finding sheds new light on the intricate behavior between Ca^2+^ ions and membranes, important for understanding the function of membranes.

## Introduction

Ca^2+^ ions are exceptionally important for the functioning of any living cell. They regulate contraction of muscles, nerve conduction, and clotting of blood cells (1,2,3,4,5,6), among other things. To do so, the Ca^2+^ ion concentration inside and outside cells needs to be carefully regulated. Within a typical human cell, the concentration of Ca^2+^ ions is maintained at 10^−4^ mM, while the concentration outside of the cell is typically 2.2 mM^3^. In order to maintain and regulate this concentration imbalance, multiple ion channels and transporters are involved in controlling the in- and outflux of Ca^2+^ ions. At the same time, to allow for full Ca^2+^ control by ion channels, the cell membrane should act as an impermeable barrier for Ca^2+^ ions. This is especially true for neurons since nerve cell operation critically depends on controllable Ca^2+^ ion in- and outflux. Therefore, it has been assumed that Ca^2+^ ions do not penetrate through lipid bilayer membranes of any kind.

Recent research has provided deeper insights into the complex role that lipid membranes play. Instead of being merely a structurally time constant barrier, the diversity and dynamics of membrane composition and its direct relation to specific diseases (7) suggests that lipid membranes fulfill many more functions than are currently known. For example, polyunsaturated phospholipids are abundant in the cell membranes of the brain and have been recently found to facilitate membrane vesiculation without leakage (8,9) as well as membrane fusion (10).

Molecular-level interfacial probes have also helped to gain more knowledge on membrane molecular structure and function. Second harmonic (SH) imaging has long been recognized as an ideal tool for probing membranes (11,12,13) since the symmetry selection rule that governs the production of SH photons ensures that, in isotropic media, only interfacial structures are measured. Another consequence of this symmetry consideration is that identical leaflets placed in opposite configurations, such as in a perfectly symmetric bilayer, will not generate a SH response. This means that SH generation is in principle an extremely sensitive probe for measuring changes in interfacial membrane structure and permeation of large molecules (14,15). However, since the process relies on nonlinear optical interactions that are intrinsically weak, all previous SH imaging experiments have been performed using resonant enhancement (16,17,18). As a consequence, it was not possible to study asymmetry in membrane hydration. Thanks to the recent invention of high-throughput SH imaging, which demonstrated a throughput increase by a factor of 5000 over standard multiphoton confocal imaging methods (19,20), it was shown that nonresonant interfacial water responses could be imaged on the subsecond timescale (21).

Subsequently, high-throughput dynamic SH imaging was applied to freely suspended lipid membranes in aqueous solution. The SH contrast was shown to arise from oriented water molecules at a charged membrane interface, and this can be used to generate spatiotemporal surface potential maps (22). Lipid bilayer membranes were shown to exhibit large spatiotemporal fluctuations in both the membrane water structure and membrane potential (23). The short-lived membrane potential fluctuations reach values of approximately −300 mV (23,24), which might be high enough to generate a transient pore, similar to the process of electroporation (25). Therefore, recent findings call into question the picture of lipid membranes being uniform impenetrable barriers for Ca^2+^ ions.

Here, we revisit the question of Ca^2+^ ion translocation by imaging the water structure at the interface of giant unilamellar vesicles (GUVs) in contact with CaCl_2_ solution using high-throughput wide-field SH imaging. We first demonstrate the power of noninvasive SH imaging by visualizing the structural hydration asymmetry of unstained lipid membranes in the form of GUVs. Since the interaction of Ca^2+^ ions directly impacts the structure of the membrane water, and SH imaging specifically measures the interfacial hydration, this provides a unique and direct way of probing the interaction. The GUVs are composed of symmetric charged bilayer membranes with a different hydrophobic barrier: fully saturated branched DPhPC:DPhPA phospholipids (1,2-diphytanoyl-sn-glycero-3-phosphocholine, 1,2-diphytanoyl-sn-glycero-3-phosphate), mono-unsaturated DOPC:DOPA lipids (1,2-dioleoyl-sn-glycero-3-phosphocholine, 1,2-dioleoyl-sn-glycero-3-phosphate) with and without cholesterol, and poly-unsaturated SLPC:SLPA lipids (1-stearoyl-2-linoleoyl-sn-glycero-3-phosphocholine, 1-stearoyl-2-linoleoyl-sn-glycero-3-phosphate). Polyunsaturated lipids are abundant in the plasma membranes of brain cells (8,9,26), and their interaction with Ca^2+^ is therefore of great importance. Surprisingly, we observe Ca^2+^ translocation for the mono-unsaturated (DOPC:DOPA) lipid membrane (with an average translocation time of 22–30 *μ*s/ion across the whole GUV surface, which corresponds to a permeability coefficient of ∼10^−8^ cm/s). Translocation does not happen homogeneously and varies threefold along the GUV surface. Adding cholesterol reduces the rate of translocation (to 150–200 *μ*s/ion). Complete inhibition of translocation is observed for the poly-unsaturated SLPC:SLPA membrane, as well as for the branched lipid membrane (DPhPC:DPhPA). The observed results were supplemented by all-atom molecular dynamics simulations, where we showed that the free-energy cost of transient pore formation follows the same order of membrane composition. The lower density of the hydrophobic core of the DOPC:DOPA membrane favors pore formation compared with the branched and polyunsaturated lipids membranes. Formation of transmembrane pores is enhanced due to the presence of transmembrane potential induced by asymmetric distribution of Ca^2+^ ions. Once the ions are translocated, two-photon fluorescence (2PF) measurements show that they stay at the membrane. These results shed new light on the importance of lipids and, in particular, suggest another functionality for poly-unsaturated lipids, namely to control Ca^2+^ in- and outflux. Likewise, they suggest that the membrane may also play a role as a Ca^2+^ reservoir.

## Materials and methods

### Chemicals and cleaning procedures

1,2-diphytanoyl-sn-glycero-3-phosphocholine (DPhPC), 1,2-diphytanoyl-sn-glycero-3- phosphate (DPhPA), 1,2-dioleoyl-sn-glycero-3-phosphocholine (DOPC), 1,2-dioleoyl-snglycero-3-phosphate (DOPA), 1-stearoyl-2-linoleoyl-sn-glycero-3-phosphocholine (SLPC), 1- stearoyl-2-linoleoyl-sn-glycero-3-phosphate (SLPA) and cholesterol in powder form (>99%) were purchased from Avanti Polar Lipids. CaCl_2_ (99.999%), poly(vinyl alcohol) (PVA, Mw 146000 - 186000, >99%), bovine serum albumin (BSA, > 99%), glucose, sucrose, and chloroform (>99.8%) were purchased from Sigma-Aldrich. The Ca^2+^ sensitive dye Fluo-4 (Pentapotassium Salt, cell impermeant) was purchased from Thermo-Fisher Scientific. All chemicals were used as received. All aqueous solutions were made with ultra-pure water (H2O, Milli-Q UF plus, Millipore, Inc., electrical resistance of 18.2 MΩ cm). All aqueous solutions were filtered with 0.1 µM Millex filters. The coverslips used in the imaging were precleaned with piranha solution (1:3 - 30% H_2_O_2_: 95-97% H_2_SO_4_) and thoroughly rinsed with ultrapure water.

### PVA-assisted GUV growth and transfer

GUVs were formed by gel-assisted growth using polyvinyl alcohol (PVA) (see supporting material S1 and S2 for more details). Lipids dissolved in chloroform (5–10 µl, 1 mg/ml) were then deposited on a dried PVA film and the chamber was placed under vacuum for 30 min. The growth chamber was filled with a solution composed of 30 or 45 mM sucrose in order to match the osmolarity of the observation solution. After the desired vesicle sizes were reached, the GUVs were transferred into the observation chamber using a pipette. An open observation chamber was assembled separately using a cleaned coverslip, coated with bovine serum albumin (BSA) and rinsed with ultrapure water. It was then placed inside the SH microscope and filled with an observation solution composed of 30 mM glucose and 5 mM CaCl_2_.

### Wide-field SH microscopy

SH images were obtained with a custom built wide-field second harmonic microscope. The microscope is pumped by either femtosecond laser source (Femtolux 3, 1030 nm, 1 MHz, 220 fs) or custom built optical parametric amplifier based on Femtolux 3 (670 - 1000 nm, 1 MHz, 23 - 50 fs). Combination of a lens (f = 25 cm, Thorlabs) and 60x water immersion objective lens (Olympus LUMPFLN 60XW, NA 1.0) allows the laser beam to excite an area of 90 µm on a sample plane at normal incidence angle. SH light is collected in a forward direction with a 60x objective lens (Olympus LUMFLN 60XW, NA 1.1) and imaged into an electronically amplified intensified CCD camera (EM-ICCD, PiMax-4, Princeton Instruments) with an 18 cm tube lens. A 750 nm short pass filter (FESH0750, Thorlabs) and a 515 nm band pass filter (FL514.5-10) were used in the detection path to get rid of the fundamental beam. The lateral resolution of the microscope is 400 nm. For polarization control a half-wave plate for the fundamental beam and a combination of a half-wave plate and a Glan–Taylor prism for detected light were used. For white-light imaging, the sample is illuminated from the top using a white light source and the linear scattered light is detected in the forward direction with the same objective lens.

## Results and discussion

### SH imaging of interfacial water of GUVs

GUVs were formed from a mixture of zwitterionic and anionic lipids by PVA-assisted swelling in a 45 mM sucrose solution, followed by a gentle transfer into an observation chamber with a glucose solution of matching osmolarity (27,28,29) (see supporting material S1 and S2 for more details on PVA-assisted GUV growth and transfer). This procedure was applied to produce GUVs with the following compositions: DOPC:DOPA, DPhPC:DPhPA, SLPC:SLPA with a 1:1 M ratio, and DOPC:DOPA:Chol with a 2:2:1 M ratio. These compositions were chosen to exemplify different structural motifs in cellular membranes. Branched lipids form dense membranes (30), and lipids with two unsaturated chains form more open structures (8,31,32), while cholesterol is known to increase the density of the membrane hydrophobic core (33,34,35,36). Lipids with a saturated alkyl chain in combination with an alkyl chain that has two unsaturated bonds pack more densely and should therefore have a higher hydrophobic energy barrier for ion translocation. All these structural motifs are found in eukaryotic membranes, but the importance of the latter type of lipids has received attention only recently (7,9).

**Figure 1:**
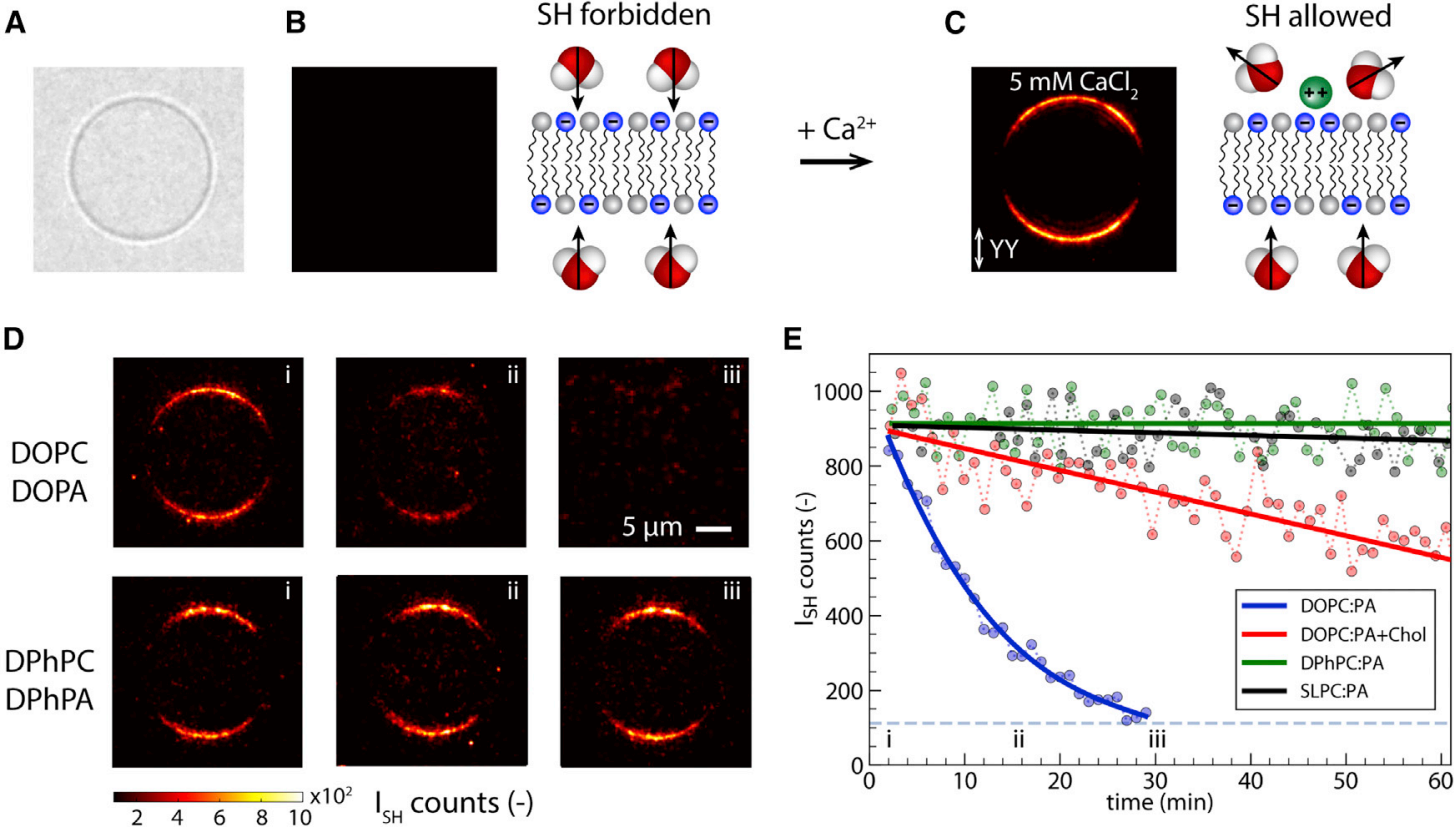
SH imaging of interfacial water of GUVs. (*A*) White-light image of a single GUV. (*B*) SH image of a symmetric charged DPhPC:DPhPA 1:1 GUV in a glucose/sucrose solution, together with a schematic description of the symmetric interface. (*C*) SH image of the same GUV after adding 5 mM CaCl_2_ to the surrounding solution together with a schematic description of the asymmetry induced by the addition of ions. The GUV was imaged with vertical polarization with an acquisition time of 0.5 s and 20 exposures averaged. (*D*) SH images of GUVs taken over time composed of DOPC:DOPA 1:1 (*top*) and DPhPC:DPhPA 1:1 (*bottom*) with added 5 mM CaCl_2_ to the outside solution. Scale bar is 5 *μ*m, the magnification of all images is equal. (*E*) Integrated SH signal generated by a single GUV over time for DOPC:DOPA 1:1 (*blue*), DOPC:DOPA:Chol 2:2:1 (*red*), SLPC:PA 1:1 (*black*), and DPhPC:DPhPA 1:1 (*green*) compositions, with t = 0 marking the addition of ions into the outside solution (*dots*: data points, *solid lines*: a guide for the eye, *horizontal dashed line*: noise level). At least 10 different vesicles were observed across three independently prepared samples for each set of conditions.

The thus-prepared GUVs are precipitated onto a coverslip and imaged with a home-built wide-field SH microscope (19,20). In brief, a collimated femtosecond laser beam creates a 90 *μ*m illumination area on the sample plane and illuminates the entire GUV with X or Y polarized laser pulses. SH photons are collected in the phase-matching direction with a 60× objective (numerical aperture = 1.1) and detected with an EM-ICCD camera (see supporting material S3 for more details about the microscope). SH light is collected from the equatorial plane of the GUV. Fig. 1 *A* shows a white-light image of a GUV composed of a symmetric charged DPhPC:DPhPA bilayer with a 1:1 mol ratio. The corresponding SH image shown in Fig. 1 *B* has no detectable SH contrast. Since the bilayer and the adjacent water are fully symmetric, SH emission is forbidden within the electric dipole approximation.

When 5 mM CaCl_2_ salt is added to the solution outside of the GUV, SH contrast emerges as shown in Fig. 1 *C*. Here, Ca^2+^ ions bind with the charged headgroups of the DPhPA lipids, which changes the orientation of the interfacial water on the outside of the GUV and therefore breaks the initial centrosymmetry. This modification alters the number of noncentrosymmetrically distributed water molecules across the membrane but not the distribution of lipids. Additionally, in terms of number density, oriented water molecules outnumber lipid molecules by a factor of ∼100 (37,38). Taking this all into account, we conclude that the SH intensity arises from oriented water molecules, which are only present at the inner leaflet of the GUV when the electrostatic potential on the outer leaflet has vanished. The images shown in Fig. 1 are recorded with vertical polarization labeled YY, where the first and second letter indicate the polarization of the SH light and the fundamental light, respectively. The change in averaged intensity along the GUV outline is caused by the projection of the electromagnetic field vector onto the interface, which generates a $cos^{2}\left(\theta \right)$ dependence. Domains of brighter and darker areas are seen along the GUV outline with a size of 1.5–3 *μ*m, similar to observations on planar free-standing bilayers (23). Within these short-lived domains of lipid-water-ion complexes, the surface potential can reach values of up to ∼ −300 mV (23).

### Ca^2+^ translocation

We performed SH imaging of a variety of lipid GUV membranes that have the same PC and PA headgroups in a 1:1 mol ratio but different alkyl chains: DOPC:DOPA, DOPC:DOPA:Chol, DPhPC:DPhPA, and SLPC:SLPA. GUVs from these different lipids were imaged repeatedly (period t = 1 min and accumulation time 5 s) after adding 5 mM CaCl_2_ into the outside solution. Fig. 1 *D* shows SH images recorded in YY polarization for DOPC:DOPA and DPhPC:DPhPA GUVs right after the addition of salt (t = 0 min, column i), after t = 15 min (ii), and after t = 30 min (iii), respectively. Fig. 1 *E* shows the integrated intensity as a function of time for the four different membranes. The SH intensity for the DOPC:DOPA membrane decreases monotonously and vanishes after 1800 s. Such behavior takes place across all GUVs in the sample, and 1 h after adding salt, it is no longer possible to find a GUV with a detectable SH signal across more than 100 vesicles per sample. Adding cholesterol slows down the decay of the SH intensity on average by a factor of ∼10. Changing the unsaturation in the alkyl chains from two mono-unsaturated chains to one saturated and one doubly unsaturated chain as in the SLPC:SLPA membrane results in constant average SH intensity. The DPhPC:DPhPA membrane with saturated branched alkyl chains displays the same behavior. At least 10 different vesicles were observed across three independently prepared samples for each set of conditions, with at least three vesicles observed per sample.

**Figure 2:**
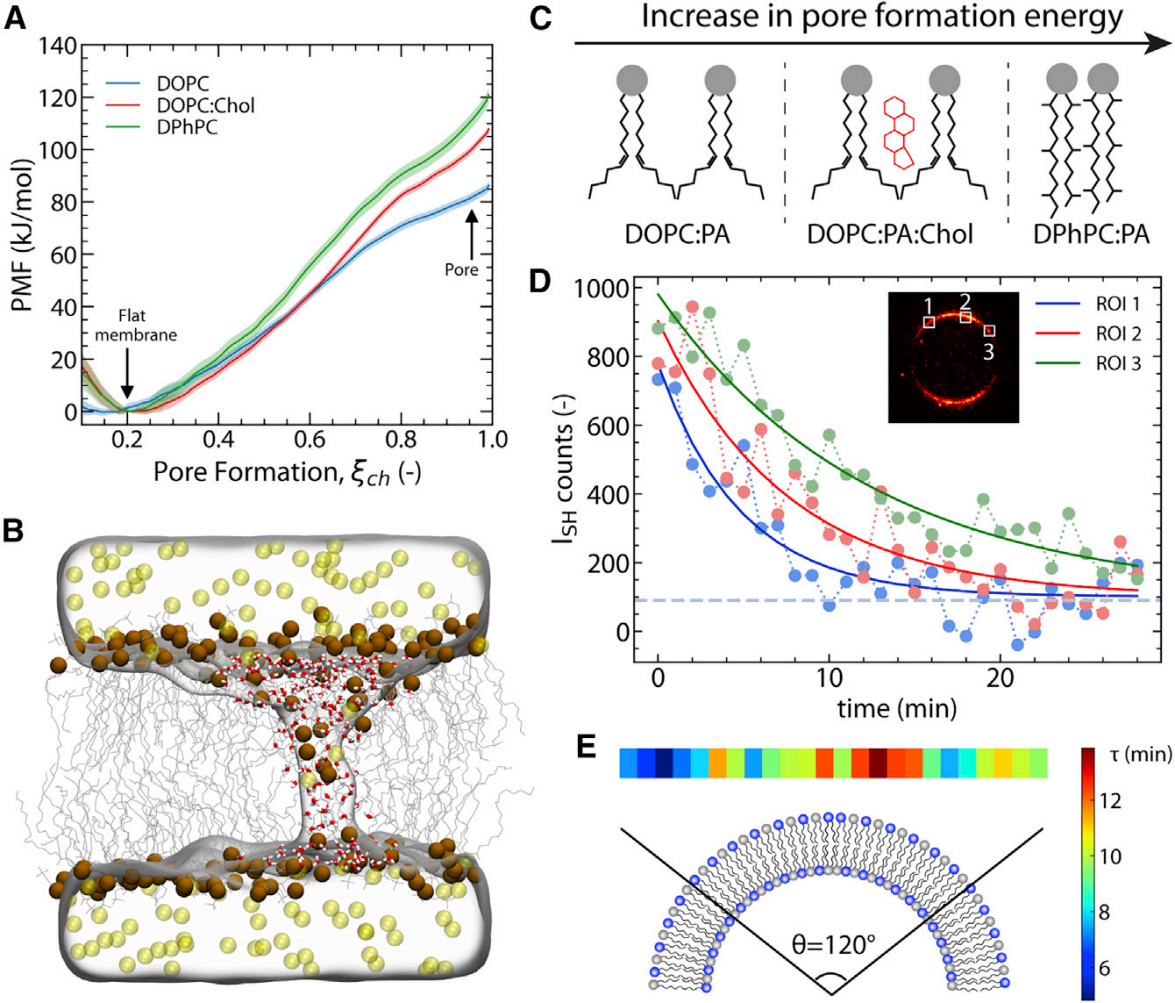
Translocation of Ca^2+^ through various membranes. (*A*) Potentials of mean force (PMFs) of pore formation over lipid membranes with different compositions (see legend) at a transmembrane voltage of −300 mV. (*B*) Simulation snapshot of a DOPC membrane with an open pore; Ca^2+^ ions are rendered as yellow spheres, lipid phosphorus atoms as brown spheres, and water molecules inside the membrane as sticks, with water additionally rendered as a transparent surface. (*C*) The alkyl chains of different lipid compositions used in this work and their configuration in the bilayer. (*D*) Decay curves of SH intensity for 3 different ROIs (*dots*) of DOPC:DOPA 1:1 GUV shown in the inset fitted with an exponential decay (*solid line*). (*E*) Map of decay time constants along the DOPC:DOPA 1:1 GUV surface for the central part of the GUV. At least 10 different vesicles were observed across three independently prepared samples for each set of conditions.

The vanishing of SH intensity following the addition of Ca^2+^ to the solution can only arise from a restoration of centrosymmetry, i.e., the molecular structure of water on both interfaces is mirror imageable in the membrane plane. This can only be achieved if the Ca^2+^-water-lipid structure is, on average, identical on both leaflets, which in turn can only occur if the Ca^2+^ translocates through the membrane. The GUVs were observed under white-light imaging before and after the experiment. Since the starting SH intensities of all four mixtures are comparable, we can assume that the density of Ca^2+^ ions is determined by the headgroups only. We hypothesize that the transmembrane potential induced by binding of Ca^2+^ ions to anionic membranes is high enough to form short-lived transmembrane pores in unsaturated membranes (39). Through those pores, Ca^2+^ ions can translocate to the other side and thus restore the centrosymmetry of the bilayer. To verify this hypothesis, we performed all-atom molecular dynamics (MD) simulations to investigate the probability of pore formation for different hydrophobic motifs observed experimentally (see supporting material S4–S6 for more details on MD simulations). Fig. 2 *A* shows the computed potentials of mean force (PMFs; sometimes referred to as free-energy profile) for the formation of an aqueous defect over DOPC, DOPC:Chol, and DPhPC membranes at a transmembrane voltage of 300 mV (Fig. S2 shows the PMFs for different applied transmembrane voltages). The PMFs were computed along the chain reaction coordinate ξ_ch_, which quantifies the connectivity of the polar transmembrane defect (40). It was previously shown that PMF calculations along ξ_ch_ do not suffer from hysteresis problems, in contrast to several other reaction coordinates for pore formation, and that free-energy barriers are not integrated out (41,42,43). The values ξ_ch_ = 0.2 and ξ_ch_ ≈ 0.95 correspond to the states of the flat membrane and the open pore, respectively. Fig. 2 *B* shows a snapshot from MD simulations of an open pore through which Ca^2+^ ions translocate from one side of the membrane to the other. Fig. S3 shows additional snapshots of other membrane compositions. The PMFs reveal that while there is a considerable free-energy cost for forming a pore in all the three membranes, there is a strong influence from the lipid composition. Namely, the formation of transient pores is more favorable in unsaturated DOPC, while the presence of cholesterol or saturated branched tails significantly decreases the probability of pore formation. This is schematically shown in Fig. 2 *C*. Notably, the reduced free energy of pore formation over the DOPC membrane is further enhanced with increasing transmembrane potential, demonstrating that DOPC membranes are more sensitive to transmembrane potentials compared with membranes containing cholesterol or branched tails (Fig. S2). To test whether the transient pores enable rapid Ca^2+^ flux over the membrane, as required for a decay of the SH intensity, we carried out additional simulations with open pores by restraining the systems to ξ_ch_ = 1 and an applied constant potential of 600 mV (Fig. S4). At a high simulated concentration of 1000 mM Ca^2+^, we observed ∼660 Ca^2+^ permeations per microsecond across the porous DOPC membrane. This value translates into a single-pore permeability (flux per concentration) of ∼10^−12^ cm^3^/s, indicative of a highly conducting defect.

This facilitated pore formation over the DOPC membrane can be intuitively understood as the free energy of pore formation being strongly controlled by the head-to-tail volume ratio of the lipids. Generally, inverted cone-shaped lipids with large head-to-tail volume ratios favor pore formation because their positive intrinsic curvature matches with the geometry along the pore rim; by the same token, cone-shaped lipids disfavor pore formation (44). In addition, the membrane thickness is a critical parameter, as the free-energy cost of pore nucleation strongly increases with membrane thickness (44,45). In the DPhPC:DPhPA mixture, the bulky branched tails impose a small head-to-tail volume ratio and hence favor negative curvature, which is incompatible with the large positive curvature along the pore rim. The addition of cholesterol, with its small hydroxyl headgroup and bulky polycyclic tail, has a similar effect. In addition, cholesterol renders the membrane thicker and more rigid, which increases the cost of the formation of a transmembrane defect. Keeping the number of unsaturated bonds identical but moving them on one alkyl chain as in an SLPC:SLPA mixture, the bilayer becomes thicker and the tails more ordered, which may explain the lower Ca^2+^ permeability compared with DOPC:DOPA.

There is a good qualitative agreement between experimental observations and simulations. Thus, the observed SH signal decrease can be attributed to the translocation of ions through transient pores in lipid membranes induced by membrane potential. By directly SH imaging the interfacial water of GUV membranes, it is immediately clear that Ca^2+^ ions do translocate through lipid bilayer membranes via transient pores in a hydrophobic core-dependent manner (as one would expect).

We can now use the known headgroup charge density of anionic lipid membranes (σ = −1 mC/m^2^) (23) as well as the typical size of GUVs (R = 10 *μ*m) to compute the translocation rate of Ca^2+^ ions. Using this number, the average translocation time per Ca^2+^ across the whole GUV surface is ∼22–30 *μ*s. The average translocation time for the DOPC:DOPA:Chol mixture is ∼150–200 *μ*s. Translocation of Ca^2+^ ions through a DOPC:DOPA membrane is not homogeneous along the GUV surface. To demonstrate this, we plot the SH signal decay for three different regions of interest (ROIs) as shown in Fig. 2 *D*. We observed different decays for each ROI with time constants of 4, 13, and 9 min, respectively, obtained from fitting decays with an exponential curve (black lines). In order to visualize the spatial distribution of decays along the GUV surface, we first converted the GUV images to polar coordinates and normalized the SH signal by a $cos^{2}\left(\theta \right)$ dependence. We then used only the central part of the GUV with an acceptance angle of 120° (Fig. 2 *E*) in order to avoid regions with vanishing SH contrast. The SH intensity decay was then analyzed separately for 25 different ROIs. Fig. 2 *E* shows the map of time decays along the GUV surface, which reveals the spatially inhomogeneous translocation of Ca^2+^ ions through the lipid membrane.

To understand the apparent discrepancy with experimental evidence that suggests that Ca^2+^ ions do not penetrate membranes, we conducted the same Ca^2+^ permeation experiment as in Fig. 1 *D* using the DOPC:DOPA GUVs but added Ca^2+^ indicator dye to the inside of the GUV (46,47,48). The result, shown in Fig. S5, suggests that Ca^2+^ does not interact with the Ca^2+^ indicator dye in the bulk solution inside the GUV. At the same time, current measurements on free-standing lipid bilayers of DOPC:DOPA that were exposed to CaCl_2_ on the top leaflet did not display a change in current after CaCl_2_ was added. All three observations can be explained by taking into account the high binding constant range of Ca^2+^ ions to PC:PA lipids that was measured in the domains where binding was observed (K_D_ value range of 10^−8^–10^−11^ M) (23). These binding constants are much higher than those of Ca^2+^ indicator dyes, which reach maximum values of 325 nM (47). Thus, when Ca^2+^ is added to the solution containing the GUVs, it binds to the outer membrane. After binding, translocation can occur until a fully symmetric bilayer is generated with Ca^2+^ bound to both leaflets. These translocated Ca^2+^ ions then stay for extended periods of time in the interfacial region rather than dissociating into the aqueous phase, which means there will be neither a current nor a change in the 2PF signal from fluorophore-Ca^2+^ complexes. Employing other lipids could bring in some Ca^2+^ by translocation, but this is limited because the lipid-water-Ca^2+^ complexes are strongly bound and therefore act as a Ca^2+^ reservoir. Note that within this two-dimensional interface, the Ca^2+^ ions move along the hydrated membrane plane.

Thus, based on membrane interfacial water imaging, we find that Ca^2+^ ions can penetrate through lipid membranes and that this depends on the structure of the hydrophobic core. Formation of transmembrane pores is enhanced due to the presence of transmembrane potential induced by asymmetric distribution of Ca^2+^ ions. Poly-unsaturated lipids were found to be practically impenetrable for Ca^2+^ ions. This property, together with their ability to facilitate membrane fusion as required for synaptic signaling (10), may explain why poly-unsaturated lipids are major components in the membranes of neurons (8,9,26). Finally, a full understanding of the process requires a merging of mean field models with a molecular-level understanding, as couplings over different length- and timescales appear to be involved. For example, the Ca^2+^-rich micron-sized domains impact the structure of the membrane but could also lead to other processes such as budding or the formation of tubules.

## Conclusions

Summarizing, we revisited the question of Ca^2+^ ion translocation through lipid bilayer membranes by imaging the water structure at the interface of GUVs in contact with a CaCl_2_ solution using high-throughput wide-field SH imaging. The interaction of Ca^2+^ ions directly impacts the interfacial water structure, which leads to changes in the SH images. Varying the structural motifs of the hydrophobic cores of the bilayer membranes, different types of behavior are observed. Ca^2+^ translocation is observed through membranes composed of lipids with two mono-unsaturated alkyl chains, with an average translocation time of 22–30 *μ*s/ion across the whole GUV surface. Adding cholesterol reduces the rate of translocation to 150–200 *μ*s/ion. Complete inhibition of translocation is observed for the poly-unsaturated SLPC:SLPA membrane, as well as for the branched lipid DPhPC:DPhPA membrane. This difference is explained by the difference in the free energy that is required to open a transmembrane pore with the transmembrane potential induced by asymmetric distribution of Ca^2+^ ions. The geometry of branched and poly-unsaturated lipids disfavors pore formation compared with DOPC:DOPA membranes. Branched and poly-unsaturated lipids as well as cholesterol disfavor pore formation because their shape is incompatible with the positive curvature along the pore rim or because they may render the membrane thicker and more rigid. This result was supported by all-atom MD simulations of transmembrane pore formation in DOPC, DOPC:Chol, and DPhPC membranes. Once the ions are translocated, they stay on the membrane for extended periods of time, leading to a negative 2PF result. Translocation of Ca^2+^ ions happens inhomogeneously along the GUV surface. These results shed new light on the importance of lipids and, in particular, suggest an additional functionality for poly-unsaturated lipids, namely to control Ca^2+^ in and outflux. Likewise, they suggest that the membrane may also play a role as a Ca^2+^ reservoir (49) and support the idea (7) that lipids play a much more sophisticated regulating role than previously thought. Overall, our work indicates that lipid-dependent permeability of membranes to calcium ions should be carefully considered in studies studying signaling roles of calcium in living cells.

## Data availability

The data that support the findings of this study are available from the corresponding author upon reasonable request.

## Code availability

Custom programs created for image processing and decay analysis are available from the corresponding author upon reasonable request. A modified GROMACS version that implements the chain coordinate for pore formation is available at https://gitlab.com/cbjh/gromacs-chain-coordinate.

## Author contributions

M.E. and D.R. performed experimental measurements and reproducibility validations. C.S.P. and J.S.H. performed analytical computations and simulations. S.R., J.S.H., A.R., D.R., M.E., and C.S.P. wrote the manuscript. S.R., A.R., and J.H. supervised the work. Both M.E. and D.R. contributed equally and have the right to list their name first in their CV. All authors contributed to the article and approved the submitted version.

## Supporting material

Document S1. Supporting material and Figures S1–S5

Document S2. Article plus supporting material
