# Supplementary material for: Passive transport of Ca2+ ions through lipid bilayers imaged by widefield second harmonic microscopy
Source: Biophys J. 2023 Jan 19;122(4):624–31. doi: 10.1016/j.bpj.2023.01.018 (PMC9989880; doi:10.1016/j.bpj.2023.01.018)
Supplement: Document S1. Supporting material and Figures S1–S5 [file mmc1.pdf]

Biophysical Journal, Volume *122*

Supplemental information

Passive transport of Ca^{2+} ions through lipid bilayers imaged by wide-field second harmonic microscopy

Maksim Eremchev, David Roesel, Chetan S. Poojari, Aurélien Roux, Jochen S. Hub, and Sylvie Roke

Supplementary Information

for

Passive transport of Ca^{2+} ions through lipid bilayers imaged by wide-field second harmonic microscopy

Maksim Eremchev[a,1], David Roesel[a,1], Chetan S. Poojari[b], Aurelien Roux[c,d,e], Jochen S. Hub[b], and Sylvie Roke[a,f,g,*]

[a]Laboratory for Fundamental BioPhotonics (LBP), Institute of Bioengineering (IBI), School of Engineering (STI), École Polytechnique Fédérale de Lausanne (EPFL), CH-1015 Lausanne, Switzerland; [b]Theoretical Physics and Center for Biophysics, Saarland University, 66123 Saarbrücken, Germany; [c]Biochemistry Department, University of Geneva, Geneva, Switzerland; [d]Swiss National Centre for Competence in Research Programme Chemical Biology, Geneva, Switzerland; [e]School of Chemistry and Biochemistry, University of Geneva, Geneva, Switzerland; [f]Institute of Materials Science and Engineering (IMX), School of Engineering (STI), École Polytechnique Fédérale de Lausanne (EPFL), CH-1015 Lausanne, Switzerland; and [g]Lausanne Centre for Ultrafast Science, École Polytechnique Fédérale de Lausanne (EPFL), CH-1015 Lausanne, Switzerland.

[1]M.Y.E and D.R. contributed equally to this work.

**To whom correspondence may be addressed. Email: sylvie.roke@epfl.ch.*

Table of Contents:

S1. Chemicals and cleaning procedures
S2. PVA-assisted GUV growth and transfer
S3. Wide-field SH microscopy
S4. MD simulation setup
S5. Free energy calculations of pore formation
S6. Simulations of calcium permeation
S7. 2PF imaging of Ca^{2+} permeation

S1. Chemicals and cleaning procedures

1,2-diphytanoyl-sn-glycero-3-phosphocholine (DPhPC), 1,2-diphytanoyl-sn-glycero-3-phosphate (DPhPA), 1,2-dioleoyl-sn-glycero-3-phosphocholine (DOPC), 1,2-dioleoyl-sn-glycero-3-phosphate (DOPA), 1-stearoyl-2-linoleoyl-sn-glycero-3-phosphocholine (SLPC), 1-stearoyl-2-linoleoyl-sn-glycero-3-phosphate (SLPA) and cholesterol in powder form (>99%) were purchased from Avanti Polar Lipids. $CaCl_2$ (99.999%), poly(vinyl alcohol) (PVA, Mw 146000 - 186000, >99%), bovine serum albumin (BSA, > 99%), glucose, sucrose, and chloroform (>99.8%) were purchased from Sigma-Aldrich. The Ca^{2+} sensitive dye Fluo-4 (Pentapotassium Salt, cell impermeant) was purchased from Thermo-Fisher Scientific. All chemicals were used as received. All aqueous solutions were made with ultra-pure water (H_2O, Milli-Q UF plus, Millipore, Inc., electrical resistance of 18.2 MΩ cm). All aqueous solutions were filtered with 0.1 µM Millex filters. The coverslips used in the imaging were pre-cleaned with piranha solution (1:3 - 30% H_2O_2: 95-97% H_2SO_4) and thoroughly rinsed with ultrapure water.

Figure S1 represents structures of all lipid molecules used in this work.

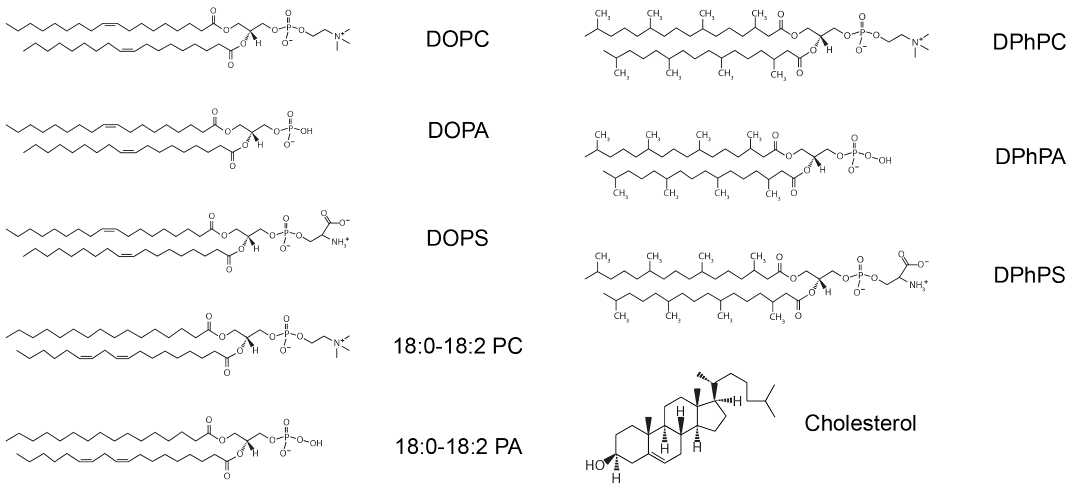

Figure S1: Structure of lipids used in this work.

S2. PVA-assisted GUV growth and transfer

Giant unilamellar vesicles were formed by gel-assisted growth using polyvinyl alcohol (PVA) similar to the process described by Weinberger et al[1]. In brief, a 5% (w/w) solution of PVA in water was prepared and heated to 90 °C in a water bath. A rubber O-ring was bonded to a cleaned circular glass coverslip using a silicone elastomer (Kwik-Cast, World Precision Instruments) to form an open growth chamber and 50 µl of heated PVA was spread on the coverslip and dried for 30 minutes at 50 °C. Lipids dissolved in chloroform (5–10 µl, 1 mg/ml)

were then deposited on the dried PVA film and the chamber was placed under vacuum for 30 min in order to evaporate the chloroform.

The growth chamber was filled with a solution composed of 30 or 45 mM sucrose (with an addition of 100 µM phosphate buffer if required) in order to match the osmolarity of the observation solution. The growth chamber was observed using a light microscope (AmScope T490) in order to track the formation of vesicles. After the desired vesicle sizes were reached, typically in < 30 min, the GUVs were transferred into the observation chamber using a pipette. Due to the difference in density of the inside and outside solution, many GUVs precipitates to the bottom of the observation chamber and could either be imaged immediately or immobilized by pipette aspiration for more time-sensitive measurements.

An open observation chamber (Quick Change Chamber, Warner Instruments) was assembled separately using a cleaned coverslip, coated with bovine serum albumin (BSA) in order to prevent rupture of GUVs coming into contact with the glass surface, and rinsed with ultrapure water. It was then placed inside the SH microscope and filled with an observation solution composed of 30 mM glucose and 5 mM $CaCl_2$. In some cases, 100 µM of phosphate buffer was added to the observation solution in order to maintain a constant relative charge of the anionic lipids.

S3. Wide-field SH microscopy

SH images were obtained with a custom built wide-field second harmonic microscope. The microscope is pumped by either femtosecond laser source (Femtolux 3, 1030 nm, 1 MHz, 220 fs) or custom built optical parametric amplifier based on Femtolux 3 (670 - 1000 nm, 1 MHz, 23 - 50 fs). Combination of a lens (f = 25 cm, Thorlabs) and 60x water immersion objective lens (Olympus LUMPFLN 60XW, NA 1.0) allows the laser beam to excite an area of 90 µm on a sample plane at normal incidence angle. SH light is collected in a forward direction with a 60x objective lens (Olympus LUMFLN 60XW, NA 1.1) and imaged into an electronically amplified intensified CCD camera (EM-ICCD, PiMax-4, Princeton Instruments) with an 18 cm tube lens. A 750 nm short pass filter (FESH0750, Thorlabs) and a 515 nm band pass filter (FL514.5-10) were used in the detection path to get rid of the fundamental beam. The lateral resolution of the microscope is 400 nm. For polarization control a half-wave plate for the fundamental beam and a combination of a half-wave plate and a Glan–Taylor prism for detected light were used. For white-light imaging, the sample is illuminated from the top using a white light source and the linear scattered light is detected in the forward direction with the same objective lens.

Each GUV was first imaged by white light microscopy, in order to exclude multilamellar vesicles or vesicles with defects. These vesicles were excluded from analysis independently,

without being observed by SH imaging, and therefore without any information about their potential brightness. After this initial screening, no further selection of vesicles was performed and each vesicle was measured by SH imaging, and subsequently analyzed.

The GUVs were generally highly stable, and the number of GUVs that collapsed on the experimental timescales was statistically insignificant. Experimental times were determined by the complete vanishing of SH signal. For example in the case of DOPC:PA, this was typically around 30 minutes. For the case of DPhPC:PA and SLPC:PA, where no permeation (and therefore no signal decay) was observed, the experimental times spanned up to 3 hours. Control measurements have also shown that neither stability nor permeation rates were influenced by laser illumination, since the observed trends remained identical even if the laser was turned off."

S4. Molecular dynamics (MD) simulation setup

MD simulation systems were set up with the MemGen webserver[2]. The systems contained 162 lipids and 40 water molecules per lipid. Lipid interactions were described with the Charmm36 force field with electronic continuum correction (ECC)[3–5]. The Charmm-modified TIP3P water model was applied[6]. Ca^{2+} and chloride parameters were taken from Ref.[7,8]. Equilibration simulations were carried out with GROMACS, version 2021[9]. Electrostatic interactions were described with the particle-mesh Ewald method[10,11]. Lennard-Jones interactions were truncated at 1.2 nm, while smoothly switching off the forces between 1.0 and 1.2 nm. The geometry of water molecules was constrained with the SETTLE algorithm[12]. The geometry of bonds involving hydrogen atoms were constrained with p-LINCS[13]. To allow the use of a 4 fs integration time step, we used hydrogen mass repartitioning (HMR)[14], as implemented by the CHARMM-GUI service[15]. The temperature was controlled at 310 K using velocity rescaling, coupling membrane and solvent to separate heat baths (τ = 0.5 ps)[16]. The pressure was kept at 1 bar using the semi-isotropic Berendsen barostat, which we used owing to its numerical stability for equilibration simulations[17]. After energy minimization, the systems were equilibrated for 100 ns.

S5. Free energy calculations of membrane pore formation

Potentials of mean force (PMFs) of pore formation were computed along the chain reaction coordinate ξ_{ch}, which quantifies the degree of connectivity of a polar defect over the lipid membrane [18,19]. The coordinate ξ_{ch}, is defined using a trans-membrane cylinder whose axis is aligned with the membrane normal (z direction) and whose center along z is is placed that membrane center of mass. The cylinder is decomposed into N_s slices and ξ_{ch} is defined as the fraction of slices that are filled by polar heavy atoms (here, oxygen atoms from water and lipid

phosphate groups). Hence, by pulling the simulation system along ξ_{ch}, the slices are filled with polar atoms one-by-one, thereby forming a continuous transmembrane polar defect. Harmonic restraints along ξ_{ch}, have been implemented into an in-house modification of GROMACS 2021, which is freely available at https://gitlab.com/cbjh/gromacs-chain-coordinate. For technical details we refer to Ref. [18]. Critically, in contrast to PMF calculations along several other reaction, PMF calculations along ξ_{ch} do not suffer from hysteresis problems and free energy barriers are not integrated out[20].

An initial pore was formed using constant-velocity pulling from ξ_{ch} = 0.1 to ξ_{ch} = 1 over 100 ns with a force constant of 3000 kJ/mol along ξ_{ch}. To define ξ_{ch}, a cylinder with radius R_{cyl} = 0.9 nm was used, and the cylinder was decomposed into slices with a thickness of d_s = 0.1 nm. The number of slices N_s, was chosen such that 20% of the slices are filled by polar atoms ($\xi_{ch} \approx 0.2$) in the unperturbed membrane. This convention led to 26, 32, and 28 slices for the membranes of DOPC, DOPC:Chol and DPhPC, respectively. Fig. S2 shows the computed PMFs of pore formation for membranes of DOPC, DOPC:Chol 80:20, and DPhPC while applying transmembrane voltages of (A) 0 mV, (B) 300 mV, and (C) 600 mV as well as free energy of pore formation for all three cases.

The PMFs were computed with umbrella sampling using 27 umbrella windows. Initial frames for umbrella sampling were taken from the constant-velocity pulling simulation. We used the following non-equidistant spacing for the umbrella reference positions: 0.065 through 0.625 in steps of 0.08, and 0.64 through 1.0 in steps of 0.02. For reference positions smaller or larger than 0.7 we used force constants of 5000 or 10000 kJ/mol, respectively. Each window was simulated for 100 ns, where the first 20 ns were omitted for equilibration. PMFs were computed with the weighted histogram analysis method (WHAM), as implemented in the gmx wham module of GROMACS[21,22]. Statistical errors were estimated using 50 rounds of bootstrapping of complete histograms[22].

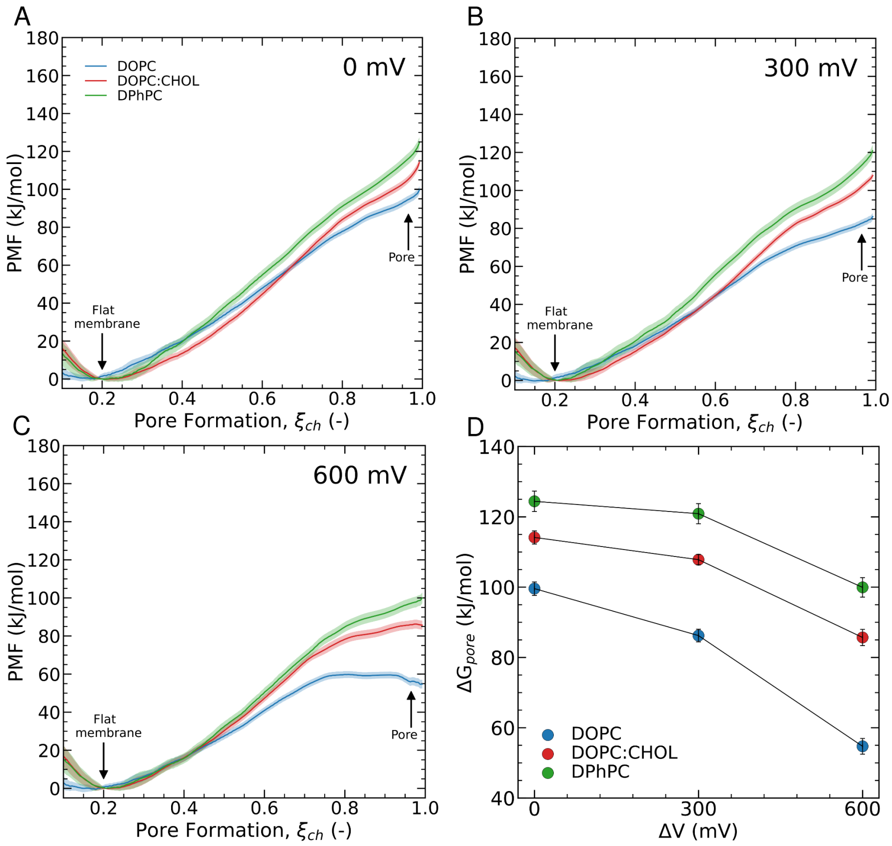

Fig. S2. PMFs of pore formation for membranes of DOPC, DOPC:Chol 80:20, and DPhPC (see legend for color code) while applying transmembrane voltages of (A) 0 mV, (B) 300 mV, and (C) 600 mV. (D) Free energy of pore formation versus transmembrane potential ΔV for the three lipid compositions.

S6. Simulations of calcium permeation across an open pore

Systems for the simulation of Ca^{2+} permeation across an open pore were set up with MemGen, with membrane patches composed of 162 DOPC lipids, 162 DPhPC lipids, 130 DOPC:32 Chol lipids, and 40 water molecules per lipid[2]. Water molecules were replaced with Ca^{2+} and Cl^- ions to obtain a concentration 1000 mM of $CaCl_2$. Hence, to obtain good permeation number statistics, we simulated a $CaCl_2$ concentration that is by far larger than physiological concentrations. The simulations were carried out with the Charmm36 lipid force field with electronic continuum correction (ECC), a Charmm36 variant with improved lipid–ion interactions[3–5]. Ca^{2+} and chloride parameters were taken from Ref.[7,8]. The temperature was controlled at 310 K using velocity rescaling, coupling membrane and solvent to separate heat baths (τ = 1 ps)[16]. The pressure was kept at 1 bar using the semi-isotropic Parrinello-Rahman barostat[23]. No HMR was applied, and an integration of 2 fs was used. Other parameters were

used as described above. The membrane patches were equilibrated for 500 ns without any restraints.

A pore was induced in the equilibrated membranes by pulling along ξ_{ch} from 0.1 to 1 over 100 ns using a force constant of 3000 kJ/mol. Then, the system was restrained at ξ_{ch} = 1 with a force constant of 10000 and simulated for 500 ns. Fig. S3 shows the simulation snapshots of membranes with an open pore for DOPC, DOPC:Chol, and DPhPC lipids. The Ca^{2+} permeation was obtained by counting the number of permeation events across the pore (in any direction, Fig. S4).

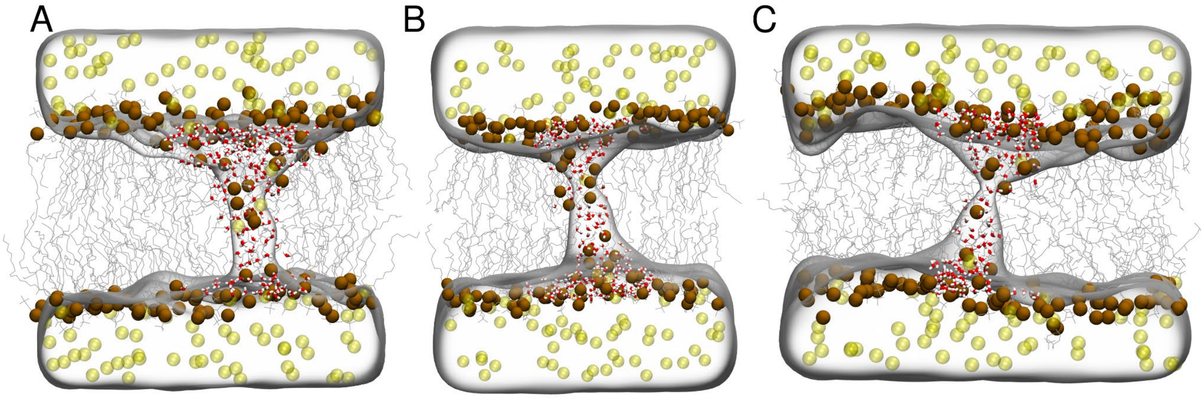

Fig. S3. Simulation snapshots of membranes with an open pore, maintained by restraining the systems to ξ_{ch} = 1, for (A) DOPC, (B) DOPC:Chol 80:20, and (C) DPhPC. Ca^{2+} ions are rendered as yellow spheres, lipid phosphorus atoms as brown spheres and water molecules inside the membrane as sticks. Water is in addition rendered as a transparent surface.

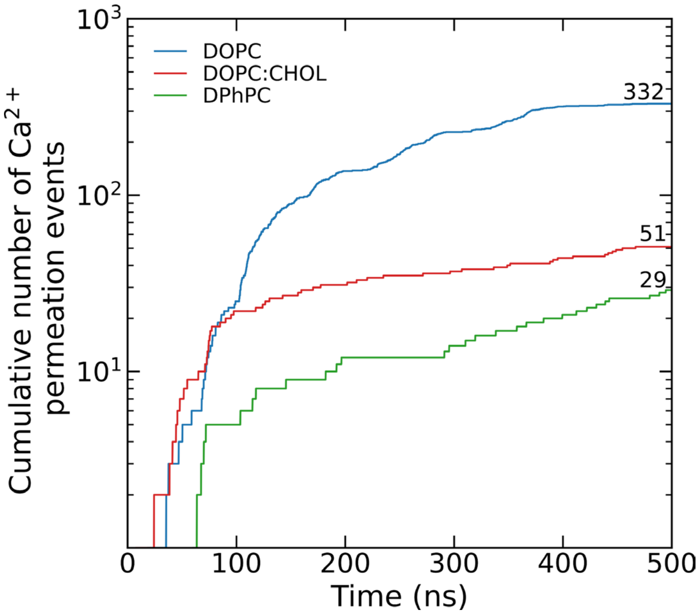

Fig. S4. Number of cumulative calcium permeation events versus simulation time for membranes at 600 mV with an open pore, maintained by restraining the systems to ξ_{ch} = 1. The increased number of permeation events for DOPC as compared to DOPC:Chol or DPhPC is rationalized by the larger pore across the DOPC membrane.

S7. 2PF imaging of Ca^{2+} permeation

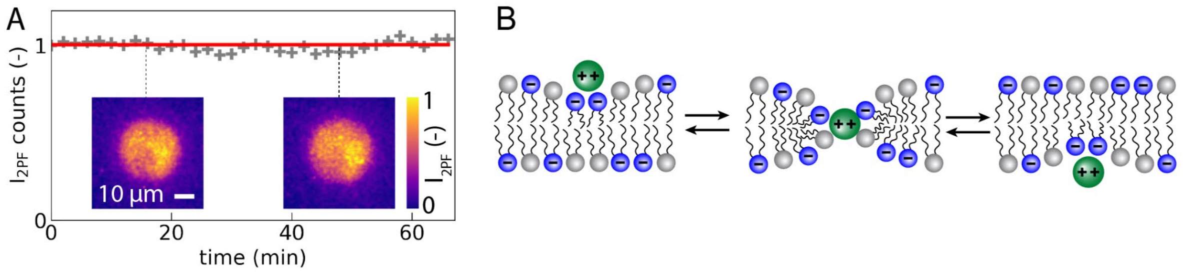

Fig. S5: Ca^{2+} permeation imaged by fluorescent probes. (A) Fluorescence signal produced by Fluo-4 inside the DOPC:DOPA 1:1 GUV over time suggesting that Ca^{2+} ions do not penetrate model lipid membranes. Inset: fluorescence images of a GUV with a Fluo-4 probe inside taken at different times after adding $CaCl_2$ to the outside solution. (B) Schematic illustration of a Ca^{2+} ion passing through the membrane and remaining at the inner interface.

References

1. Weinberger, A. *et al.* Gel-assisted formation of giant unilamellar vesicles. *Biophys. J.* **105**, 154–164 (2013).
2. Knight, C. J. & Hub, J. S. MemGen: a general web server for the setup of lipid membrane simulation systems. *Bioinformatics* **31**, 2897–2899 (2015).
3. Pastor, R. W. & MacKerell, A. D. Development of the CHARMM Force Field for Lipids. *J. Phys. Chem. Lett.* **2**, 1526–1532 (2011).
4. Melcr, J. *et al.* Accurate Binding of Sodium and Calcium to a POPC Bilayer by Effective Inclusion of Electronic Polarization. *J. Phys. Chem. B* **122**, 4546–4557 (2018).
5. Nencini, R. *et al.* Prosecco: polarization reintroduced by optimal scaling of electronic continuum correction origin in MD simulations. Available at: https://gitlab.com/sparkly/prosecco/prosECCo75. Accessed Oct 19, 2022.
6. Jorgensen, W. L., Chandrasekhar, J., Madura, J. D., Impey, R. W. & Klein, M. L. Comparison of simple potential functions for simulating liquid water. *J. Chem. Phys.* **79**, 926–935 (1983).
7. Kohagen, M., Mason, P. E. & Jungwirth, P. Accurate Description of Calcium Solvation in Concentrated Aqueous Solutions. *J. Phys. Chem. B* **118**, 7902–7909 (2014).
8. Kohagen, M., Mason, P. E. & Jungwirth, P. Accounting for Electronic Polarization Effects in Aqueous Sodium Chloride via Molecular Dynamics Aided by Neutron Scattering. *J. Phys. Chem. B* **120**, 1454–1460 (2016).
9. Abraham, M. J. *et al.* GROMACS: High performance molecular simulations through multi-level parallelism from laptops to supercomputers. *SoftwareX* **1–2**, 19–25 (2015).
10. Darden, T., York, D. & Pedersen, L. Particle mesh Ewald: An N·log(N) method for Ewald sums in large systems. *J. Chem. Phys.* **98**, 10089–10092 (1993).
11. Essmann, U. *et al.* A smooth particle mesh Ewald method. *J. Chem. Phys.* **103**, 8577–8593 (1995).
12. Miyamoto, S. & Kollman, P. A. Settle: An analytical version of the SHAKE and RATTLE algorithm for rigid water models. *J. Comput. Chem.* **13**, 952–962 (1992).

13. Hess, B. P-LINCS: A Parallel Linear Constraint Solver for Molecular Simulation. *J. Chem. Theory Comput.* **4**, 116–122 (2008).
14. Feenstra, K. A., Hess, B. & Berendsen, H. J. C. Improving efficiency of large time-scale molecular dynamics simulations of hydrogen-rich systems. *J. Comput. Chem.* **20**, 786–798 (1999).
15. Gao, Y. *et al.* CHARMM-GUI Supports Hydrogen Mass Repartitioning and Different Protonation States of Phosphates in Lipopolysaccharides. *J. Chem. Inf. Model.* **61**, 831–839 (2021).
16. Bussi, G., Donadio, D. & Parrinello, M. Canonical sampling through velocity rescaling. *J. Chem. Phys.* **126**, 014101 (2007).
17. Berendsen, H. J. C., Postma, J. P. M., van Gunsteren, W. F., DiNola, A. & Haak, J. R. Molecular dynamics with coupling to an external bath. *J. Chem. Phys.* **81**, 3684–3690 (1984).
18. Hub, J. S. & Awasthi, N. Probing a Continuous Polar Defect: A Reaction Coordinate for Pore Formation in Lipid Membranes. *J. Chem. Theory Comput.* **13**, 2352–2366 (2017).
19. Ting, C. L., Awasthi, N., Müller, M. & Hub, J. S. Metastable Prepores in Tension-Free Lipid Bilayers. *Phys. Rev. Lett.* **120**, 128103 (2018).
20. Awasthi, N. & Hub, J. S. Simulations of Pore Formation in Lipid Membranes: Reaction Coordinates, Convergence, Hysteresis, and Finite-Size Effects. *J. Chem. Theory Comput.* **12**, 3261–3269 (2016).
21. Kumar, S., Rosenberg, J. M., Bouzida, D., Swendsen, R. H. & Kollman, P. A. THE weighted histogram analysis method for free-energy calculations on biomolecules. I. The method. *J. Comput. Chem.* **13**, 1011–1021 (1992).
22. Hub, J. S., de Groot, B. L. & van der Spoel, D. g_wham—A Free Weighted Histogram Analysis Implementation Including Robust Error and Autocorrelation Estimates. *J. Chem. Theory Comput.* **6**, 3713–3720 (2010).
23. Parrinello, M. & Rahman, A. Polymorphic transitions in single crystals: A new molecular dynamics method. *J. Appl. Phys.* **52**, 7182–7190 (1981).
