# Supplementary material for: Passive transport of Ca2+ ions through lipid bilayers imaged by widefield second harmonic microscopy
Source: Biophys J. 2023 Jan 19;122(4):624–31. doi: 10.1016/j.bpj.2023.01.018 (PMC9989880; doi:10.1016/j.bpj.2023.01.018)
Supplement: Document S2. Article plus supporting material [file mmc2.pdf]

Biophysical Journal
Article

Biophysical Society

# Passive transport of $Ca^{2+}$ ions through lipid bilayers imaged by widefield second harmonic microscopy

Maksim Eremchev,[1] David Roesel,[1] Chetan S. Poojari,[2] Aurélien Roux,[3,4,5] Jochen S. Hub,[2] and Sylvie Roke[1,6,7,*]
[1]Laboratory for Fundamental BioPhotonics (LBP), Institute of Bioengineering (IBI), School of Engineering (STI), École Polytechnique Fédérale de Lausanne (EPFL), Lausanne, Switzerland; [2]Theoretical Physics and Center for Biophysics, Saarland University, Saarbrücken, Germany; [3]Biochemistry Department, University of Geneva, Geneva, Switzerland; [4]Swiss National Centre for Competence in Research Programme Chemical Biology, Geneva, Switzerland; [5]School of Chemistry and Biochemistry, University of Geneva, Geneva, Switzerland; [6]Institute of Materials Science and Engineering (IMX), School of Engineering (STI), École Polytechnique Fédérale de Lausanne (EPFL), Lausanne, Switzerland; and [7]Lausanne Centre for Ultrafast Science, École Polytechnique Fédérale de Lausanne (EPFL), Lausanne, Switzerland

ABSTRACT In biology, release of $Ca^{2+}$ ions in the cytosol is essential to trigger or control many cell functions. Calcium signaling acutely depends on lipid membrane permeability to $Ca^{2+}$. For proper understanding of membrane permeability to $Ca^{2+}$, both membrane hydration and the structure of the hydrophobic core must be taken into account. Here, we vary the hydrophobic core of bilayer membranes and observe different types of behavior in high-throughput wide-field second harmonic imaging. $Ca^{2+}$ translocation is observed through mono-unsaturated (DOPC:DOPA) membranes, reduced upon the addition of cholesterol, and completely inhibited for branched (DPhPC:DPhPA) and poly-unsaturated (SLPC:SLPA) lipid membranes. We propose, using molecular dynamics simulations, that ion transport occurs through ion-induced transient pores, which requires nonequilibrium membrane restructuring. This results in different rates at different locations and suggests that the hydrophobic structure of lipids plays a much more sophisticated regulating role than previously thought.

SIGNIFICANCE Interaction of $Ca^{2+}$ ions with lipid membranes regulates a variety of processes like membrane fusion, fission, and signaling. It is generally accepted that $Ca^{2+}$ ions do not passively permeate through pure lipid membranes of any kind. However, the impermeability to $Ca^{2+}$ ions is not compatible with recently proposed fusion and fission mechanisms, which logically predict that $Ca^{2+}$ ions should be able to translocate through membranes (albeit at low rates). Here, we revisit the question of $Ca^{2+}$ ion translocation through lipid membranes, by performing molecular interface specific measurements complemented by molecular dynamics simulations and standard fluorescence measurements and find that passive ion permeation does occur. Our finding sheds new light on the intricate behavior between $Ca^{2+}$ ions and membranes, important for understanding the function of membranes.

## INTRODUCTION

$Ca^{2+}$ ions are exceptionally important for the functioning of any living cell. They regulate contraction of muscles, nerve conduction, and clotting of blood cells (1–6), among other things. To do so, the $Ca^{2+}$ ion concentration inside and outside cells needs to be carefully regulated. Within a typical human cell, the concentration of $Ca^{2+}$ ions is maintained at $10^{-4}$ mM, while the concentration outside of the cell is typically 2.2 mM[3]. In order to maintain and regulate this concentration imbalance, multiple ion channels and transporters are involved in controlling the in- and outflux of $Ca^{2+}$ ions. At the same time, to allow for full $Ca^{2+}$ control by ion channels, the cell membrane should act as an impermeable barrier for $Ca^{2+}$ ions. This is especially true for neurons since nerve cell operation critically depends on controllable $Ca^{2+}$ ion in- and outflux. Therefore, it has been assumed that $Ca^{2+}$ ions do not penetrate through lipid bilayer membranes of any kind.

Recent research has provided deeper insights into the complex role that lipid membranes play. Instead of being merely a structurally time constant barrier, the diversity and dynamics of membrane composition and its direct relation to specific diseases (7) suggests that lipid membranes fulfill many more functions than are currently known. For example, polyunsaturated phospholipids are abundant in

*Submitted October 27, 2022, and accepted for publication January 17, 2023.*

*Correspondence: sylvie.roke@epfl.ch

Maksim Eremchev and David Roesel contributed equally to this work.

Editor: John Conboy.

https://doi.org/10.1016/j.bpj.2023.01.018

© 2023 Biophysical Society.

This is an open access article under the CC BY license (http://creativecommons.org/licenses/by/4.0/).

the cell membranes of the brain and have been recently found to facilitate membrane vesiculation without leakage (8,9) as well as membrane fusion (10).

Molecular-level interfacial probes have also helped to gain more knowledge on membrane molecular structure and function. Second harmonic (SH) imaging has long been recognized as an ideal tool for probing membranes (11–13) since the symmetry selection rule that governs the production of SH photons ensures that, in isotropic media, only interfacial structures are measured. Another consequence of this symmetry consideration is that identical leaflets placed in opposite configurations, such as in a perfectly symmetric bilayer, will not generate a SH response. This means that SH generation is in principle an extremely sensitive probe for measuring changes in interfacial membrane structure and permeation of large molecules (14,15). However, since the process relies on nonlinear optical interactions that are intrinsically weak, all previous SH imaging experiments have been performed using resonant enhancement (16–18). As a consequence, it was not possible to study asymmetry in membrane hydration. Thanks to the recent invention of high-throughput SH imaging, which demonstrated a throughput increase by a factor of 5000 over standard multiphoton confocal imaging methods (19,20), it was shown that nonresonant interfacial water responses could be imaged on the subsecond timescale (21).

Subsequently, high-throughput dynamic SH imaging was applied to freely suspended lipid membranes in aqueous solution. The SH contrast was shown to arise from oriented water molecules at a charged membrane interface, and this can be used to generate spatiotemporal surface potential maps (22). Lipid bilayer membranes were shown to exhibit large spatiotemporal fluctuations in both the membrane water structure and membrane potential (23). The short-lived membrane potential fluctuations reach values of approximately −300 mV (23,24), which might be high enough to generate a transient pore, similar to the process of electroporation (25). Therefore, recent findings call into question the picture of lipid membranes being uniform impenetrable barriers for $Ca^{2+}$ ions.

Here, we revisit the question of $Ca^{2+}$ ion translocation by imaging the water structure at the interface of giant unilamellar vesicles (GUVs) in contact with $CaCl_2$ solution using high-throughput wide-field SH imaging. We first demonstrate the power of noninvasive SH imaging by visualizing the structural hydration asymmetry of unstained lipid membranes in the form of GUVs. Since the interaction of $Ca^{2+}$ ions directly impacts the structure of the membrane water, and SH imaging specifically measures the interfacial hydration, this provides a unique and direct way of probing the interaction. The GUVs are composed of symmetric charged bilayer membranes with a different hydrophobic barrier: fully saturated branched DPhPC:DPhPA phospholipids (1,2-diphytanoyl-sn-glycero-3-phosphocholine, 1,2-diphytanoyl-sn-glycero-3-phosphate), mono-unsaturated DOPC:DOPA lipids (1,2-dioleoyl-sn-glycero-3-phosphocholine, 1,2-dioleoyl-sn-glycero-3-phosphate) with and without cholesterol, and poly-unsaturated SLPC:SLPA lipids (1-stearoyl-2-linoleoyl-sn-glycero-3-phosphocholine, 1-stearoyl-2-linoleoyl-sn-glycero-3-phosphate). Polyunsaturated lipids are abundant in the plasma membranes of brain cells (8,9,26), and their interaction with $Ca^{2+}$ is therefore of great importance. Surprisingly, we observe $Ca^{2+}$ translocation for the mono-unsaturated (DOPC:DOPA) lipid membrane (with an average translocation time of 22–30 $\mu$s/ion across the whole GUV surface, which corresponds to a permeability coefficient of $\sim 10^{-8}$ cm/s). Translocation does not happen homogeneously and varies threefold along the GUV surface. Adding cholesterol reduces the rate of translocation (to 150–200 $\mu$s/ion). Complete inhibition of translocation is observed for the poly-unsaturated SLPC:SLPA membrane, as well as for the branched lipid membrane (DPhPC:DPhPA). The observed results were supplemented by all-atom molecular dynamics simulations, where we showed that the free-energy cost of transient pore formation follows the same order of membrane composition. The lower density of the hydrophobic core of the DOPC:DOPA membrane favors pore formation compared with the branched and polyunsaturated lipids membranes. Formation of transmembrane pores is enhanced due to the presence of transmembrane potential induced by asymmetric distribution of $Ca^{2+}$ ions. Once the ions are translocated, two-photon fluorescence (2PF) measurements show that they stay at the membrane. These results shed new light on the importance of lipids and, in particular, suggest another functionality for poly-unsaturated lipids, namely to control $Ca^{2+}$ in- and outflux. Likewise, they suggest that the membrane may also play a role as a $Ca^{2+}$ reservoir.

## MATERIALS AND METHODS

### Chemicals and cleaning procedures

1,2-diphytanoyl-sn-glycero-3-phosphocholine (DPhPC), 1,2-diphytanoyl-sn-glycero-3- phosphate (DPhPA), 1,2-dioleoyl-sn-glycero-3-phosphocholine (DOPC), 1,2-dioleoyl-snglycero-3-phosphate (DOPA), 1-stearoyl-2-linoleoyl-sn-glycero-3-phosphocholine (SLPC), 1-stearoyl-2-linoleoyl-sn-glycero-3-phosphate (SLPA) and cholesterol in powder form (>99%) were purchased from Avanti Polar Lipids. $CaCl_2$ (99.999%), poly(vinyl alcohol) (PVA, Mw 146000 - 186000, >99%), bovine serum albumin (BSA, > 99%), glucose, sucrose, and chloroform (>99.8%) were purchased from Sigma-Aldrich. The $Ca^{2+}$ sensitive dye Fluo-4 (Pentapotassium Salt, cell impermeant) was purchased from Thermo-Fisher Scientific. All chemicals were used as received. All aqueous solutions were made with ultra-pure water (H2O, Milli-Q UF plus, Millipore, Inc., electrical resistance of 18.2 MΩ cm). All aqueous solutions were filtered with 0.1 μM Millex filters.

The coverslips used in the imaging were precleaned with piranha solution (1:3 - 30% $H_2O_2$: 95-97% $H_2SO_4$) and thoroughly rinsed with ultrapure water.

## PVA-assisted GUV growth and transfer

GUVs were formed by gel-assisted growth using polyvinyl alcohol (PVA) (see supporting material S1 and S2 for more details). Lipids dissolved in chloroform (5–10 μl, 1 mg/ml) were then deposited on a dried PVA film and the chamber was placed under vacuum for 30 min. The growth chamber was filled with a solution composed of 30 or 45 mM sucrose in order to match the osmolarity of the observation solution. After the desired vesicle sizes were reached, the GUVs were transferred into the observation chamber using a pipette. An open observation chamber was assembled separately using a cleaned coverslip, coated with bovine serum albumin (BSA) and rinsed with ultrapure water. It was then placed inside the SH microscope and filled with an observation solution composed of 30 mM glucose and 5 mM $CaCl_2$.

## Wide-field SH microscopy

SH images were obtained with a custom built wide-field second harmonic microscope. The microscope is pumped by either femtosecond laser source (Femtolux 3, 1030 nm, 1 MHz, 220 fs) or custom built optical parametric amplifier based on Femtolux 3 (670 - 1000 nm, 1 MHz, 23 - 50 fs). Combination of a lens (f = 25 cm, Thorlabs) and 60x water immersion objective lens (Olympus LUMPFLN 60XW, NA 1.0) allows the laser beam to excite an area of 90 μm on a sample plane at normal incidence angle. SH light is collected in a forward direction with a 60x objective lens (Olympus LUMFLN 60XW, NA 1.1) and imaged into an electronically amplified intensified CCD camera (EM-ICCD, PiMax-4, Princeton Instruments) with an 18 cm tube lens. A 750 nm short pass filter (FESH0750, Thorlabs) and a 515 nm band pass filter (FL514.5-10) were used in the detection path to get rid of the fundamental beam. The lateral resolution of the microscope is 400 nm. For polarization control a half-wave plate for the fundamental beam and a combination of a half-wave plate and a Glan–Taylor prism for detected light were used. For white-light imaging, the sample is illuminated from the top using a white light source and the linear scattered light is detected in the forward direction with the same objective lens.

# RESULTS AND DISCUSSION

## SH imaging of interfacial water of GUVs

GUVs were formed from a mixture of zwitterionic and anionic lipids by PVA-assisted swelling in a 45 mM sucrose

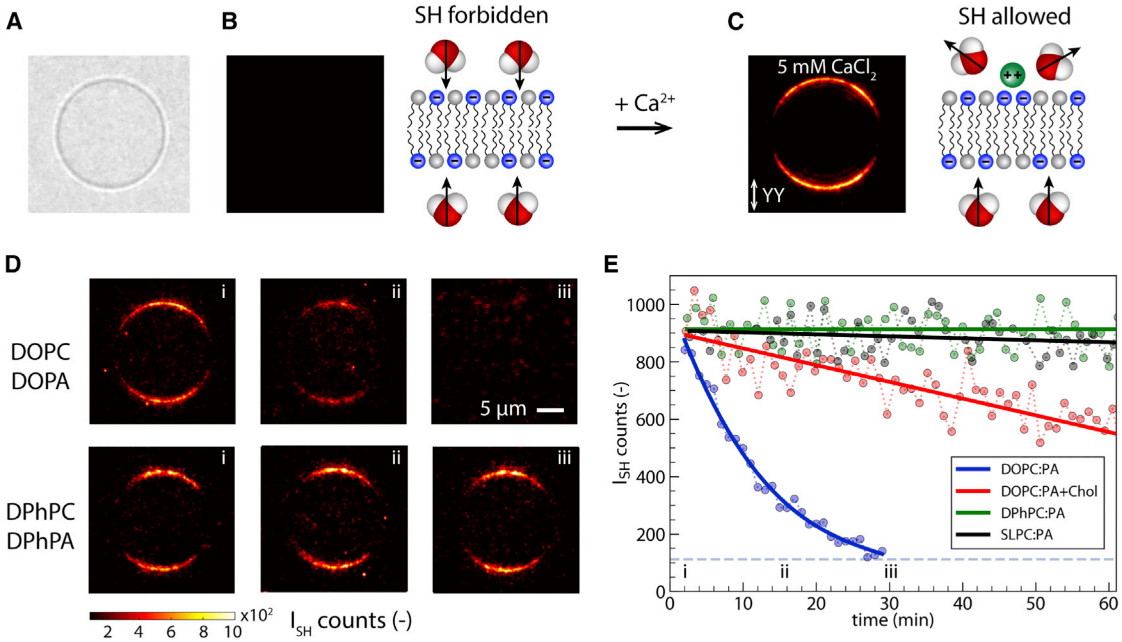


FIGURE 1 SH imaging of interfacial water of GUVs. (*A*) White-light image of a single GUV. (*B*) SH image of a symmetric charged DPhPC:DPhPA 1:1 GUV in a glucose/sucrose solution, together with a schematic description of the symmetric interface. (*C*) SH image of the same GUV after adding 5 mM $CaCl_2$ to the surrounding solution together with a schematic description of the asymmetry induced by the addition of ions. The GUV was imaged with vertical polarization with an acquisition time of 0.5 s and 20 exposures averaged. (*D*) SH images of GUVs taken over time composed of DOPC:DOPA 1:1 (*top*) and DPhPC:DPhPA 1:1 (*bottom*) with added 5 mM $CaCl_2$ to the outside solution. Scale bar is 5 μm, the magnification of all images is equal. (*E*) Integrated SH signal generated by a single GUV over time for DOPC:DOPA 1:1 (*blue*), DOPC:DOPA:Chol 2:2:1 (*red*), SLPC:PA 1:1 (*black*), and DPhPC:DPhPA 1:1 (*green*) compositions, with t = 0 marking the addition of ions into the outside solution (*dots*: data points, *solid lines*: a guide for the eye, *horizontal dashed line*: noise level). At least 10 different vesicles were observed across three independently prepared samples for each set of conditions.

solution, followed by a gentle transfer into an observation chamber with a glucose solution of matching osmolarity (27–29) (see supporting material S1 and S2 for more details on PVA-assisted GUV growth and transfer). This procedure was applied to produce GUVs with the following compositions: DOPC:DOPA, DPhPC:DPhPA, SLPC:SLPA with a 1:1 M ratio, and DOPC:DOPA:Chol with a 2:2:1 M ratio. These compositions were chosen to exemplify different structural motifs in cellular membranes. Branched lipids form dense membranes (30), and lipids with two unsaturated chains form more open structures (8,31,32), while cholesterol is known to increase the density of the membrane hydrophobic core (33–36). Lipids with a saturated alkyl chain in combination with an alkyl chain that has two unsaturated bonds pack more densely and should therefore have a higher hydrophobic energy barrier for ion translocation. All these structural motifs are found in eukaryotic membranes, but the importance of the latter type of lipids has received attention only recently (7,9).

The thus-prepared GUVs are precipitated onto a coverslip and imaged with a home-built wide-field SH microscope (19,20). In brief, a collimated femtosecond laser beam creates a 90 $\mu$m illumination area on the sample plane and illuminates the entire GUV with X or Y polarized laser pulses. SH photons are collected in the phase-matching direction with a 60× objective (numerical aperture = 1.1) and detected with an EM-ICCD camera (see supporting material S3 for more details about the microscope). SH light is collected from the equatorial plane of the GUV. Fig. 1 *A* shows a white-light image of a GUV composed of a symmetric charged DPhPC:DPhPA bilayer with a 1:1 mol ratio. The corresponding SH image shown in Fig. 1 *B* has no detectable SH contrast. Since the bilayer and the adjacent water are fully symmetric, SH emission is forbidden within the electric dipole approximation.

When 5 mM $CaCl_2$ salt is added to the solution outside of the GUV, SH contrast emerges as shown in Fig. 1 *C*. Here, $Ca^{2+}$ ions bind with the charged headgroups of the DPhPA lipids, which changes the orientation of the interfacial water on the outside of the GUV and therefore breaks the initial centrosymmetry. This modification alters the number of noncentrosymmetrically distributed water molecules across the membrane but not the distribution of lipids. Additionally, in terms of number density, oriented water molecules outnumber lipid molecules by a factor of ~100 (37,38). Taking this all into account, we conclude that the SH intensity arises from oriented water molecules, which are only present at the inner leaflet of the GUV when the electrostatic potential on the outer leaflet has vanished. The images shown in Fig. 1 are recorded with vertical polarization labeled YY, where the first and second letter indicate the polarization of the SH light and the fundamental light, respectively. The change in averaged intensity along the GUV outline is caused by the projection of the electromagnetic field vector onto the interface, which generates a $cos^2(\theta)$ dependence. Domains of brighter and darker areas are seen along the GUV outline with a size of 1.5–3 $\mu$m, similar to observations on planar free-standing bilayers (23). Within

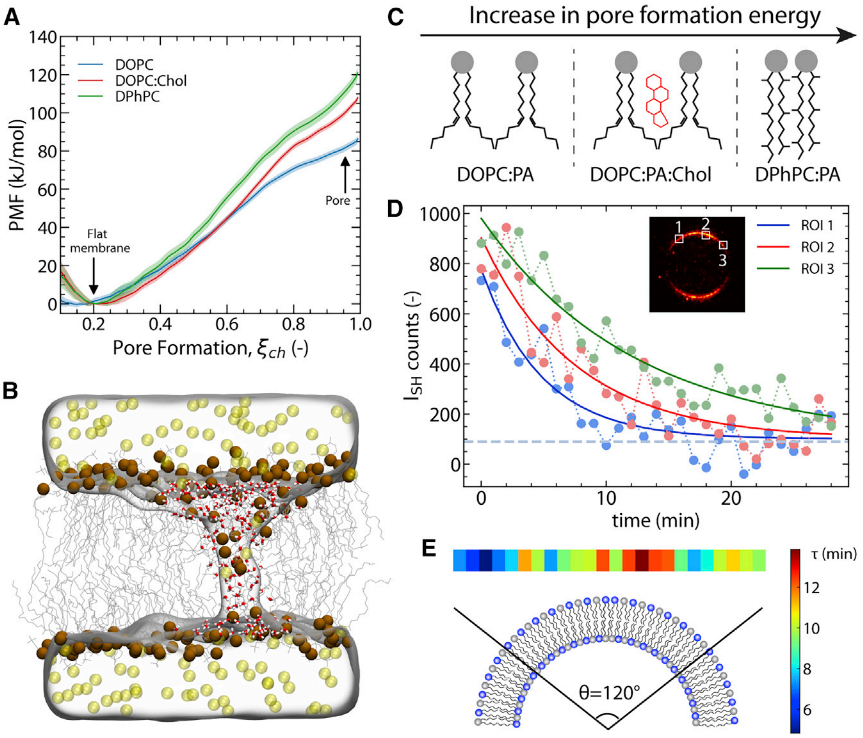


FIGURE 2 Translocation of $Ca^{2+}$ through various membranes. (*A*) Potentials of mean force (PMFs) of pore formation over lipid membranes with different compositions (see legend) at a transmembrane voltage of −300 mV. (*B*) Simulation snapshot of a DOPC membrane with an open pore; $Ca^{2+}$ ions are rendered as yellow spheres, lipid phosphorus atoms as brown spheres, and water molecules inside the membrane as sticks, with water additionally rendered as a transparent surface. (*C*) The alkyl chains of different lipid compositions used in this work and their configuration in the bilayer. (*D*) Decay curves of SH intensity for 3 different ROIs (*dots*) of DOPC:DOPA 1:1 GUV shown in the inset fitted with an exponential decay (*solid line*). (*E*) Map of decay time constants along the DOPC:DOPA 1:1 GUV surface for the central part of the GUV. At least 10 different vesicles were observed across three independently prepared samples for each set of conditions.

these short-lived domains of lipid-water-ion complexes, the surface potential can reach values of up to $\sim -300$ mV (23).

## $Ca^{2+}$ translocation

We performed SH imaging of a variety of lipid GUV membranes that have the same PC and PA headgroups in a 1:1 mol ratio but different alkyl chains: DOPC:DOPA, DOPC:DOPA:Chol, DPhPC:DPhPA, and SLPC:SLPA. GUVs from these different lipids were imaged repeatedly (period t = 1 min and accumulation time 5 s) after adding 5 mM $CaCl_2$ into the outside solution. Fig. 1 *D* shows SH images recorded in YY polarization for DOPC:DOPA and DPhPC:DPhPA GUVs right after the addition of salt (t = 0 min, column i), after t = 15 min (ii), and after t = 30 min (iii), respectively. Fig. 1 *E* shows the integrated intensity as a function of time for the four different membranes. The SH intensity for the DOPC:DOPA membrane decreases monotonously and vanishes after 1800 s. Such behavior takes place across all GUVs in the sample, and 1 h after adding salt, it is no longer possible to find a GUV with a detectable SH signal across more than 100 vesicles per sample. Adding cholesterol slows down the decay of the SH intensity on average by a factor of ~10. Changing the unsaturation in the alkyl chains from two mono-unsaturated chains to one saturated and one doubly unsaturated chain as in the SLPC:SLPA membrane results in constant average SH intensity. The DPhPC:DPhPA membrane with saturated branched alkyl chains displays the same behavior. At least 10 different vesicles were observed across three independently prepared samples for each set of conditions, with at least three vesicles observed per sample.

The vanishing of SH intensity following the addition of $Ca^{2+}$ to the solution can only arise from a restoration of centrosymmetry, i.e., the molecular structure of water on both interfaces is mirror imageable in the membrane plane. This can only be achieved if the $Ca^{2+}$-water-lipid structure is, on average, identical on both leaflets, which in turn can only occur if the $Ca^{2+}$ translocates through the membrane. The GUVs were observed under white-light imaging before and after the experiment. Since the starting SH intensities of all four mixtures are comparable, we can assume that the density of $Ca^{2+}$ ions is determined by the headgroups only. We hypothesize that the transmembrane potential induced by binding of $Ca^{2+}$ ions to anionic membranes is high enough to form short-lived transmembrane pores in unsaturated membranes (39). Through those pores, $Ca^{2+}$ ions can translocate to the other side and thus restore the centrosymmetry of the bilayer. To verify this hypothesis, we performed all-atom molecular dynamics (MD) simulations to investigate the probability of pore formation for different hydrophobic motifs observed experimentally (see supporting material S4–S6 for more details on MD simulations). Fig. 2 *A* shows the computed potentials of mean force (PMFs; sometimes referred to as free-energy profile) for the formation of an aqueous defect over DOPC, DOPC:Chol, and DPhPC membranes at a transmembrane voltage of 300 mV (Fig. S2 shows the PMFs for different applied transmembrane voltages). The PMFs were computed along the chain reaction coordinate $\xi_{ch}$, which quantifies the connectivity of the polar transmembrane defect (40). It was previously shown that PMF calculations along $\xi_{ch}$ do not suffer from hysteresis problems, in contrast to several other reaction coordinates for pore formation, and that free-energy barriers are not integrated out (41–43). The values $\xi_{ch} = 0.2$ and $\xi_{ch} \approx 0.95$ correspond to the states of the flat membrane and the open pore, respectively. Fig. 2 *B* shows a snapshot from MD simulations of an open pore through which $Ca^{2+}$ ions translocate from one side of the membrane to the other. Fig. S3 shows additional snapshots of other membrane compositions. The PMFs reveal that while there is a considerable free-energy cost for forming a pore in all the three membranes, there is a strong influence from the lipid composition. Namely, the formation of transient pores is more favorable in unsaturated DOPC, while the presence of cholesterol or saturated branched tails significantly decreases the probability of pore formation. This is schematically shown in Fig. 2 *C*. Notably, the reduced free energy of pore formation over the DOPC membrane is further enhanced with increasing transmembrane potential, demonstrating that DOPC membranes are more sensitive to transmembrane potentials compared with membranes containing cholesterol or branched tails (Fig. S2). To test whether the transient pores enable rapid $Ca^{2+}$ flux over the membrane, as required for a decay of the SH intensity, we carried out additional simulations with open pores by restraining the systems to $\xi_{ch} = 1$ and an applied constant potential of 600 mV (Fig. S4). At a high simulated concentration of 1000 mM $Ca^{2+}$, we observed ~660 $Ca^{2+}$ permeations per microsecond across the porous DOPC membrane. This value translates into a single-pore permeability (flux per concentration) of $\sim 10^{-12}$ $cm^3$/s, indicative of a highly conducting defect.

This facilitated pore formation over the DOPC membrane can be intuitively understood as the free energy of pore formation being strongly controlled by the head-to-tail volume ratio of the lipids. Generally, inverted cone-shaped lipids with large head-to-tail volume ratios favor pore formation because their positive intrinsic curvature matches with the geometry along the pore rim; by the same token, cone-shaped lipids disfavor pore formation (44). In addition, the membrane thickness is a critical parameter, as the free-energy cost of pore nucleation strongly increases with membrane thickness (44,45). In the DPhPC:DPhPA mixture, the bulky branched tails impose a small head-to-tail volume ratio and hence favor negative curvature, which is incompatible with the large positive curvature along the pore rim. The addition of cholesterol, with its small hydroxyl headgroup and bulky polycyclic tail, has a similar effect. In addition, cholesterol renders the membrane thicker and more rigid, which increases the cost of the formation of a

transmembrane defect. Keeping the number of unsaturated bonds identical but moving them on one alkyl chain as in an SLPC:SLPA mixture, the bilayer becomes thicker and the tails more ordered, which may explain the lower $Ca^{2+}$ permeability compared with DOPC:DOPA.

There is a good qualitative agreement between experimental observations and simulations. Thus, the observed SH signal decrease can be attributed to the translocation of ions through transient pores in lipid membranes induced by membrane potential. By directly SH imaging the interfacial water of GUV membranes, it is immediately clear that $Ca^{2+}$ ions do translocate through lipid bilayer membranes via transient pores in a hydrophobic core-dependent manner (as one would expect).

We can now use the known headgroup charge density of anionic lipid membranes ($\sigma = -1$ mC/m$^2$) (23) as well as the typical size of GUVs (R = 10 $\mu$m) to compute the translocation rate of $Ca^{2+}$ ions. Using this number, the average translocation time per $Ca^{2+}$ across the whole GUV surface is ~22–30 $\mu$s. The average translocation time for the DOPC:DOPA:Chol mixture is ~150–200 $\mu$s. Translocation of $Ca^{2+}$ ions through a DOPC:DOPA membrane is not homogeneous along the GUV surface. To demonstrate this, we plot the SH signal decay for three different regions of interest (ROIs) as shown in Fig. 2 *D*. We observed different decays for each ROI with time constants of 4, 13, and 9 min, respectively, obtained from fitting decays with an exponential curve (black lines). In order to visualize the spatial distribution of decays along the GUV surface, we first converted the GUV images to polar coordinates and normalized the SH signal by a $cos^2(\theta)$ dependence. We then used only the central part of the GUV with an acceptance angle of 120° (Fig. 2 *E*) in order to avoid regions with vanishing SH contrast. The SH intensity decay was then analyzed separately for 25 different ROIs. Fig. 2 *E* shows the map of time decays along the GUV surface, which reveals the spatially inhomogeneous translocation of $Ca^{2+}$ ions through the lipid membrane.

To understand the apparent discrepancy with experimental evidence that suggests that $Ca^{2+}$ ions do not penetrate membranes, we conducted the same $Ca^{2+}$ permeation experiment as in Fig. 1 *D* using the DOPC:DOPA GUVs but added $Ca^{2+}$ indicator dye to the inside of the GUV (46–48). The result, shown in Fig. S5, suggests that $Ca^{2+}$ does not interact with the $Ca^{2+}$ indicator dye in the bulk solution inside the GUV. At the same time, current measurements on free-standing lipid bilayers of DOPC:DOPA that were exposed to $CaCl_2$ on the top leaflet did not display a change in current after $CaCl_2$ was added. All three observations can be explained by taking into account the high binding constant range of $Ca^{2+}$ ions to PC:PA lipids that was measured in the domains where binding was observed ($K_D$ value range of $10^{-8}$–$10^{-11}$ M) (23). These binding constants are much higher than those of $Ca^{2+}$ indicator dyes, which reach maximum values of 325 nM (47). Thus, when $Ca^{2+}$ is added to the solution containing the GUVs, it binds to the outer membrane. After binding, translocation can occur until a fully symmetric bilayer is generated with $Ca^{2+}$ bound to both leaflets. These translocated $Ca^{2+}$ ions then stay for extended periods of time in the interfacial region rather than dissociating into the aqueous phase, which means there will be neither a current nor a change in the 2PF signal from fluorophore-$Ca^{2+}$ complexes. Employing other lipids could bring in some $Ca^{2+}$ by translocation, but this is limited because the lipid-water-$Ca^{2+}$ complexes are strongly bound and therefore act as a $Ca^{2+}$ reservoir. Note that within this two-dimensional interface, the $Ca^{2+}$ ions move along the hydrated membrane plane.

Thus, based on membrane interfacial water imaging, we find that $Ca^{2+}$ ions can penetrate through lipid membranes and that this depends on the structure of the hydrophobic core. Formation of transmembrane pores is enhanced due to the presence of transmembrane potential induced by asymmetric distribution of $Ca^{2+}$ ions. Poly-unsaturated lipids were found to be practically impenetrable for $Ca^{2+}$ ions. This property, together with their ability to facilitate membrane fusion as required for synaptic signaling (10), may explain why poly-unsaturated lipids are major components in the membranes of neurons (8,9,26). Finally, a full understanding of the process requires a merging of mean field models with a molecular-level understanding, as couplings over different length- and timescales appear to be involved. For example, the $Ca^{2+}$-rich micron-sized domains impact the structure of the membrane but could also lead to other processes such as budding or the formation of tubules.

## CONCLUSIONS

Summarizing, we revisited the question of $Ca^{2+}$ ion translocation through lipid bilayer membranes by imaging the water structure at the interface of GUVs in contact with a $CaCl_2$ solution using high-throughput wide-field SH imaging. The interaction of $Ca^{2+}$ ions directly impacts the interfacial water structure, which leads to changes in the SH images. Varying the structural motifs of the hydrophobic cores of the bilayer membranes, different types of behavior are observed. $Ca^{2+}$ translocation is observed through membranes composed of lipids with two mono-unsaturated alkyl chains, with an average translocation time of 22–30 $\mu$s/ion across the whole GUV surface. Adding cholesterol reduces the rate of translocation to 150–200 $\mu$s/ion. Complete inhibition of translocation is observed for the poly-unsaturated SLPC:SLPA membrane, as well as for the branched lipid DPhPC:DPhPA membrane. This difference is explained by the difference in the free energy that is required to open a transmembrane pore with the transmembrane potential induced by asymmetric distribution of $Ca^{2+}$ ions. The geometry of branched and poly-unsaturated lipids disfavors pore formation compared with DOPC:DOPA membranes. Branched and poly-unsaturated lipids as well as cholesterol disfavor pore formation because their shape is incompatible

with the positive curvature along the pore rim or because they may render the membrane thicker and more rigid. This result was supported by all-atom MD simulations of transmembrane pore formation in DOPC, DOPC:Chol, and DPhPC membranes. Once the ions are translocated, they stay on the membrane for extended periods of time, leading to a negative 2PF result. Translocation of $Ca^{2+}$ ions happens inhomogeneously along the GUV surface. These results shed new light on the importance of lipids and, in particular, suggest an additional functionality for poly-unsaturated lipids, namely to control $Ca^{2+}$ in and outflux. Likewise, they suggest that the membrane may also play a role as a $Ca^{2+}$ reservoir (49) and support the idea (7) that lipids play a much more sophisticated regulating role than previously thought. Overall, our work indicates that lipid-dependent permeability of membranes to calcium ions should be carefully considered in studies studying signaling roles of calcium in living cells.

## DATA AVAILABILITY

The data that support the findings of this study are available from the corresponding author upon reasonable request.

## CODE AVAILABILITY

Custom programs created for image processing and decay analysis are available from the corresponding author upon reasonable request. A modified GROMACS version that implements the chain coordinate for pore formation is available at https://gitlab.com/cbjh/gromacs-chain-coordinate.

## SUPPORTING MATERIAL

Supporting material can be found online at https://doi.org/10.1016/j.bpj.2023.01.018.

## AUTHOR CONTRIBUTIONS

M.E. and D.R. performed experimental measurements and reproducibility validations. C.S.P. and J.S.H. performed analytical computations and simulations. S.R., J.S.H., A.R., D.R., M.E., and C.S.P. wrote the manuscript. S.R., A.R., and J.H. supervised the work. Both M.E. and D.R. contributed equally and have the right to list their name first in their CV. All authors contributed to the article and approved the submitted version.

## ACKNOWLEDGMENTS

We thank Saranya Pullanchery, Adai Colom Diego, and Hilton B. de Aguiar for useful discussions. We thank Hector Martinez-Seara for sharing Charmm36-ECC force field lipid parameter files. This work is supported by the Julia Jacobi Foundation; the Swiss National Science Foundation (grant 200021-182606-1); the European Union's Horizon 2020 research and innovation program under Marie Skłodowska-Curie grant agreement 721766 (FBI); and European Research Council grant agreement no. 951324 (H2020, R2-tension). C.S.P. and J.S.H. were supported by the Deutsche Forschungsgemeinschaft (grant no. SFB 1027/B7).

## DECLARATION OF INTERESTS

The authors declare that they have no competing interests.

## REFERENCES

1. Österberg, R. 1974. Origins of metal ions in biology. *Nature.* 249:382–383.
2. Berridge, M. J. 2012. Calcium signalling remodelling and disease. *Biochem. Soc. Trans.* 40:297–309.
3. Carafoli, E., and C. B. Klee. 1999. Calcium as a Cellular Regulator. Oxford University Press.
4. Brini, M., T. Calì, …, E. Carafoli. 2013. Intracellular calcium homeostasis and signaling. *In* Metallomics and the Cell. 119–168. L. Banci, ed Springer Netherlands.
5. Fomina, A. F., C. M. Fanger, …, M. D. Cahalan. 2000. Single channel properties and regulated expression of $Ca^{2+}$ release-activated $Ca^{2+}$ (CRAC) channels in human T cells. *J. Cell Biol.* 150:1435–1444.
6. Kavalali, E. T. 2015. The mechanisms and functions of spontaneous neurotransmitter release. *Nat. Rev. Neurosci.* 16:5–16.
7. Harayama, T., and H. Riezman. 2018. Understanding the diversity of membrane lipid composition. *Nat. Rev. Mol. Cell Biol.* 19:281–296.
8. Manni, M. M., M. L. Tiberti, …, B. Antonny. 2018. Acyl chain asymmetry and polyunsaturation of brain phospholipids facilitate membrane vesiculation without leakage. *Elife.* 7:e34394.
9. Pinot, M., S. Vanni, …, H. Barelli. 2014. Polyunsaturated phospholipids facilitate membrane deformation and fission by endocytic proteins. *Science.* 345:693–697.
10. Poojari, C. S., K. C. Scherer, and J. S. Hub. 2021. Free energies of membrane stalk formation from a lipidomics perspective. *Nat. Commun.* 12:6594.
11. Moreaux, L., O. Sandre, …, J. Mertz. 2000. Membrane imaging by simultaneous second-harmonic generation and two-photon microscopy. *Opt. Lett.* 25:320–322.
12. Sly, K. L., T. T. Nguyen, and J. C. Conboy. 2012. Lens-less surface second harmonic imaging. *Opt Express.* 20:21953–21967.
13. Roesel, D., M. Eremchev, …, S. Roke. 2022. Water as a contrast agent to quantify surface chemistry and physics using second harmonic scattering and imaging: a perspective. *Appl. Phys. Lett.* 120:160501.
14. Chen, S.-L., Y. Z. Liang, …, Q. Yuan. 2019. Simple physics in and easy manipulating of the interfacial behavior of charged molecules on drug delivery vesicles. *Mater. Today Phys.* 9:100092.
15. Srivastava, A., and K. B. Eisenthal. 1998. Kinetics of molecular transport across a liposome bilayer. *Chem. Phys. Lett.* 292:345–351.
16. Boyd, R. 2008. Nonlinear Optics, 3rd Edition. https://www.elsevier.com/books/nonlinear-optics/boyd/978-0-12-369470-6.
17. Nguyen, T. T., and J. C. Conboy. 2011. High-throughput screening of drug–lipid membrane interactions via counter-propagating second harmonic generation imaging. *Anal. Chem.* 83:5979–5988.
18. Pons, T., L. Moreaux, …, J. Mertz. 2003. Mechanisms of membrane potential sensing with second-harmonic generation microscopy. *J. Biomed. Opt.* 8:428–431.
19. Macias-Romero, C., M. E. P. Didier, …, S. Roke. 2014. High throughput second harmonic imaging for label-free biological applications. *Opt Express.* 22:31102–31112.
20. Macias-Romero, C., I. Nahalka, …, S. Roke. 2017. Optical imaging of surface chemistry and dynamics in confinement. *Science.* 357:784–788.
21. Tarun, O. B., M. Y. Eremchev, …, S. Roke. 2019. Spatiotemporal imaging of water in operating voltage-gated ion channels reveals the slow motion of interfacial ions. *Nano Lett.* 19:7608–7613.
22. Tarun, O. B., C. Hannesschläger, …, S. Roke. 2018. Label-free and charge-sensitive dynamic imaging of lipid membrane hydration on millisecond time scales. *Proc. Natl. Acad. Sci. USA.* 115:4081–4086. https://doi.org/10.1073/pnas.1719347115.

23. Tarun, O. B., H. I. Okur, …, S. Roke. 2020. Transient domains of ordered water induced by divalent ions lead to lipid membrane curvature fluctuations. *Commun. Chem.* 3:17.

24. Roesel, D., M. Eremchev, …, S. Roke. 2022. Ion-induced transient potential fluctuations facilitate pore formation and cation transport through lipid membranes. *J. Am. Chem. Soc.* 144:23352–23357.

25. Karal, M. A. S., M. K. Ahamed, …, S. Ahammed. 2020. Influence of cholesterol on electroporation in lipid membranes of giant vesicles. *Eur. Biophys. J.* 49:361–370.

26. Skotland, T., and K. Sandvig. 2019. The role of PS 18:0/18:1 in membrane function. *Nat. Commun.* 10:2752.

27. Walde, P., K. Cosentino, …, P. Stano. 2010. Giant vesicles: preparations and applications. *Chembiochem.* 11:848–865.

28. Rideau, E., F. R. Wurm, and K. Landfester. 2019. Self-assembly of giant unilamellar vesicles by film hydration methodologies. *Adv. Biosyst.* 3:1800324.

29. Weinberger, A., F. C. Tsai, …, C. Marques. 2013. Gel-assisted formation of giant unilamellar vesicles. *Biophys. J.* 105:154–164.

30. Shinoda, W., M. Mikami, …, M. Hato. 2003. Molecular dynamics study on the effect of chain branching on the physical properties of lipid bilayers: structural stability. *J. Phys. Chem. B.* 107:14030–14035.

31. Jurak, M. 2013. Thermodynamic aspects of cholesterol effect on properties of phospholipid monolayers: Langmuir and Langmuir–blodgett monolayer study. *J. Phys. Chem. B.* 117:3496–3502.

32. McConnell, H. M., and A. Radhakrishnan. 2003. Condensed complexes of cholesterol and phospholipids. *Biochim. Biophys. Acta.* 1610:159–173.

33. Hannesschlaeger, C., A. Horner, and P. Pohl. 2019. Intrinsic membrane permeability to small molecules. *Chem. Rev.* 119:5922–5953.

34. Hung, W.-C., M.-T. Lee, …, H. W. Huang. 2007. The condensing effect of cholesterol in lipid bilayers. *Biophys. J.* 92:3960–3967.

35. Róg, T., M. Pasenkiewicz-Gierula, …, M. Karttunen. 2009. Ordering effects of cholesterol and its analogues. *Biochim. Biophys. Acta.* 1788:97–121.

36. Zocher, F., D. van der Spoel, …, J. S. Hub. 2013. Local partition coefficients govern solute permeability of cholesterol-containing membranes. *Biophys. J.* 105:2760–2770.

37. Lütgebaucks, C., C. Macias-Romero, and S. Roke. 2017. Characterization of the interface of binary mixed DOPC:DOPS liposomes in water: the impact of charge condensation. *J. Chem. Phys.* 146:044701.

38. Lütgebaucks, C., G. Gonella, and S. Roke. 2016. Optical label-free and model-free probe of the surface potential of nanoscale and microscopic objects in aqueous solution. *Phys. Rev. B.* 94:195410.

39. Melikov, K. C., V. A. Frolov, …, L. V. Chernomordik. 2001. Voltage-induced nonconductive pre-pores and metastable single pores in unmodified planar lipid bilayer. *Biophys. J.* 80:1829–1836.

40. Hub, J. S., and N. Awasthi. 2017. Probing a continuous polar defect: a reaction coordinate for pore formation in lipid membranes. *J. Chem. Theor. Comput.* 13:2352–2366.

41. Awasthi, N., and J. S. Hub. 2016. Simulations of pore formation in lipid membranes: reaction coordinates, convergence, hysteresis, and finite-size effects. *J. Chem. Theor. Comput.* 12:3261–3269.

42. M. L. Berkowitz, ed 2019. Biomembrane Simulations: Computational Studies of Biological Membranes, 1st ed CRC Press.

43. Awasthi, N., and J. S. Hub. 2019. Free-energy calculations of pore formation in lipid membranes. *In* Biomembrane Simulations. 109–124. M. L. Berkowitz, ed CRC Press, Taylor & Francis Group.

44. Ting, C. L., N. Awasthi, …, J. S. Hub. 2018. Metastable prepores in tension-free lipid bilayers. *Phys. Rev. Lett.* 120:128103.

45. Bennett, W. F. D., N. Sapay, and D. P. Tieleman. 2014. Atomistic simulations of pore formation and closure in lipid bilayers. *Biophys. J.* 106:210–219.

46. Graber, Z. T., Z. Shi, and T. Baumgart. 2017. Cations induce shape remodeling of negatively charged phospholipid membranes. *Phys. Chem. Chem. Phys.* 19:15285–15295.

47. Gee, K. R., K. A. Brown, …, I. Johnson. 2000. Chemical and physiological characterization of Fluo-4 $Ca^{2+}$-indicator dyes. *Cell Calcium.* 27:97–106.

48. Bennett, I. M., H. M. V. Farfano, …, D. Gust. 2002. Active transport of $Ca^{2+}$ by an artificial photosynthetic membrane. *Nature.* 420:398–401.

49. Melcrová, A., S. Pokorna, …, L. Cwiklik. 2016. The complex nature of calcium cation interactions with phospholipid bilayers. *Sci. Rep.* 6:38035.

**Biophysical Journal, Volume *122***

## Supplemental information

# Passive transport of $Ca^{2+}$ ions through lipid bilayers imaged by wide-field second harmonic microscopy

**Maksim Eremchev, David Roesel, Chetan S. Poojari, Aurélien Roux, Jochen S. Hub, and Sylvie Roke**

# Supplementary Information

for

# Passive transport of $Ca^{2+}$ ions through lipid bilayers imaged by wide-field second harmonic microscopy

Maksim Eremchev[a,1], David Roesel[a,1], Chetan S. Poojari[b], Aurelien Roux[c,d,e], Jochen S. Hub[b], and Sylvie Roke[a,f,g,*]

[a]Laboratory for Fundamental BioPhotonics (LBP), Institute of Bioengineering (IBI), School of Engineering (STI), École Polytechnique Fédérale de Lausanne (EPFL), CH-1015 Lausanne, Switzerland; [b]Theoretical Physics and Center for Biophysics, Saarland University, 66123 Saarbrücken, Germany; [c]Biochemistry Department, University of Geneva, Geneva, Switzerland; [d]Swiss National Centre for Competence in Research Programme Chemical Biology, Geneva, Switzerland; [e]School of Chemistry and Biochemistry, University of Geneva, Geneva, Switzerland; [f]Institute of Materials Science and Engineering (IMX), School of Engineering (STI), École Polytechnique Fédérale de Lausanne (EPFL), CH-1015 Lausanne, Switzerland; and [g]Lausanne Centre for Ultrafast Science, École Polytechnique Fédérale de Lausanne (EPFL), CH-1015 Lausanne, Switzerland.

*[1]M.Y.E and D.R. contributed equally to this work.*

**To whom correspondence may be addressed. Email: sylvie.roke@epfl.ch.*

**Table of Contents:**

S1. Chemicals and cleaning procedures
S2. PVA-assisted GUV growth and transfer
S3. Wide-field SH microscopy
S4. MD simulation setup
S5. Free energy calculations of pore formation
S6. Simulations of calcium permeation
S7. 2PF imaging of $Ca^{2+}$ permeation

## S1. Chemicals and cleaning procedures

1,2-diphytanoyl-sn-glycero-3-phosphocholine (DPhPC), 1,2-diphytanoyl-sn-glycero-3-phosphate (DPhPA), 1,2-dioleoyl-sn-glycero-3-phosphocholine (DOPC), 1,2-dioleoyl-sn-glycero-3-phosphate (DOPA), 1-stearoyl-2-linoleoyl-sn-glycero-3-phosphocholine (SLPC), 1-stearoyl-2-linoleoyl-sn-glycero-3-phosphate (SLPA) and cholesterol in powder form (>99%) were purchased from Avanti Polar Lipids. $CaCl_2$ (99.999%), poly(vinyl alcohol) (PVA, Mw 146000 - 186000, >99%), bovine serum albumin (BSA, > 99%), glucose, sucrose, and chloroform (>99.8%) were purchased from Sigma-Aldrich. The $Ca^{2+}$ sensitive dye Fluo-4 (Pentapotassium Salt, cell impermeant) was purchased from Thermo-Fisher Scientific. All chemicals were used as received. All aqueous solutions were made with ultra-pure water ($H_2O$, Milli-Q UF plus, Millipore, Inc., electrical resistance of 18.2 MΩ cm). All aqueous solutions were filtered with 0.1 µM Millex filters. The coverslips used in the imaging were pre-cleaned with piranha solution (1:3 - 30% $H_2O_2$: 95-97% $H_2SO_4$) and thoroughly rinsed with ultrapure water.

Figure S1 represents structures of all lipid molecules used in this work.

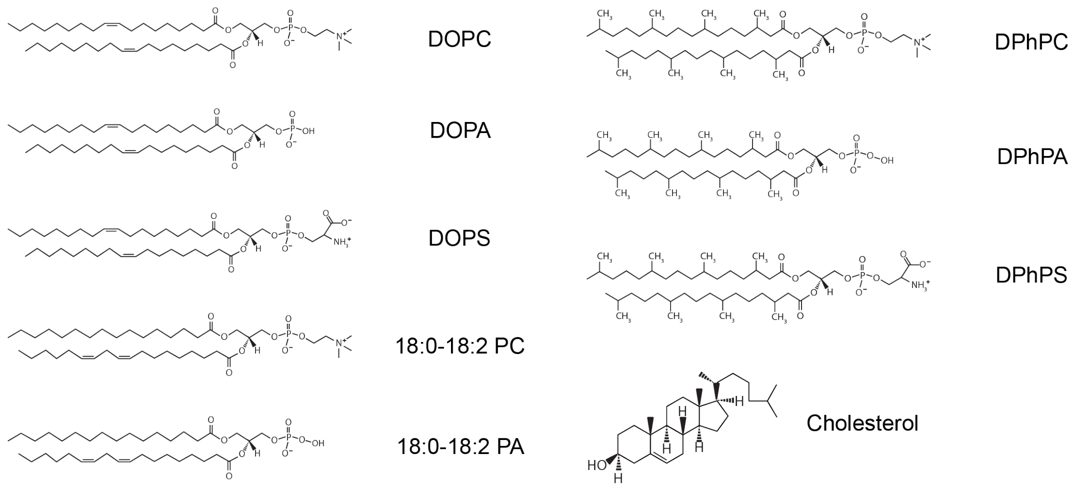


**Figure S1**: Structure of lipids used in this work.

## S2. PVA-assisted GUV growth and transfer

Giant unilamellar vesicles were formed by gel-assisted growth using polyvinyl alcohol (PVA) similar to the process described by Weinberger et al[1]. In brief, a 5% (w/w) solution of PVA in water was prepared and heated to 90 °C in a water bath. A rubber O-ring was bonded to a cleaned circular glass coverslip using a silicone elastomer (Kwik-Cast, World Precision Instruments) to form an open growth chamber and 50 µl of heated PVA was spread on the coverslip and dried for 30 minutes at 50 °C. Lipids dissolved in chloroform (5–10 µl, 1 mg/ml)

were then deposited on the dried PVA film and the chamber was placed under vacuum for 30 min in order to evaporate the chloroform.

The growth chamber was filled with a solution composed of 30 or 45 mM sucrose (with an addition of 100 µM phosphate buffer if required) in order to match the osmolarity of the observation solution. The growth chamber was observed using a light microscope (AmScope T490) in order to track the formation of vesicles. After the desired vesicle sizes were reached, typically in < 30 min, the GUVs were transferred into the observation chamber using a pipette. Due to the difference in density of the inside and outside solution, many GUVs precipitates to the bottom of the observation chamber and could either be imaged immediately or immobilized by pipette aspiration for more time-sensitive measurements.

An open observation chamber (Quick Change Chamber, Warner Instruments) was assembled separately using a cleaned coverslip, coated with bovine serum albumin (BSA) in order to prevent rupture of GUVs coming into contact with the glass surface, and rinsed with ultrapure water. It was then placed inside the SH microscope and filled with an observation solution composed of 30 mM glucose and 5 mM $CaCl_2$. In some cases, 100 µM of phosphate buffer was added to the observation solution in order to maintain a constant relative charge of the anionic lipids.

## S3. Wide-field SH microscopy

SH images were obtained with a custom built wide-field second harmonic microscope. The microscope is pumped by either femtosecond laser source (Femtolux 3, 1030 nm, 1 MHz, 220 fs) or custom built optical parametric amplifier based on Femtolux 3 (670 - 1000 nm, 1 MHz, 23 - 50 fs). Combination of a lens (f = 25 cm, Thorlabs) and 60x water immersion objective lens (Olympus LUMPFLN 60XW, NA 1.0) allows the laser beam to excite an area of 90 µm on a sample plane at normal incidence angle. SH light is collected in a forward direction with a 60x objective lens (Olympus LUMFLN 60XW, NA 1.1) and imaged into an electronically amplified intensified CCD camera (EM-ICCD, PiMax-4, Princeton Instruments) with an 18 cm tube lens. A 750 nm short pass filter (FESH0750, Thorlabs) and a 515 nm band pass filter (FL514.5-10) were used in the detection path to get rid of the fundamental beam. The lateral resolution of the microscope is 400 nm. For polarization control a half-wave plate for the fundamental beam and a combination of a half-wave plate and a Glan–Taylor prism for detected light were used. For white-light imaging, the sample is illuminated from the top using a white light source and the linear scattered light is detected in the forward direction with the same objective lens.

Each GUV was first imaged by white light microscopy, in order to exclude multilamellar vesicles or vesicles with defects. These vesicles were excluded from analysis independently,

without being observed by SH imaging, and therefore without any information about their potential brightness. After this initial screening, no further selection of vesicles was performed and each vesicle was measured by SH imaging, and subsequently analyzed.

The GUVs were generally highly stable, and the number of GUVs that collapsed on the experimental timescales was statistically insignificant. Experimental times were determined by the complete vanishing of SH signal. For example in the case of DOPC:PA, this was typically around 30 minutes. For the case of DPhPC:PA and SLPC:PA, where no permeation (and therefore no signal decay) was observed, the experimental times spanned up to 3 hours. Control measurements have also shown that neither stability nor permeation rates were influenced by laser illumination, since the observed trends remained identical even if the laser was turned off."

## S4. Molecular dynamics (MD) simulation setup

MD simulation systems were set up with the MemGen webserver[2]. The systems contained 162 lipids and 40 water molecules per lipid. Lipid interactions were described with the Charmm36 force field with electronic continuum correction (ECC)[3–5]. The Charmm-modified TIP3P water model was applied[6]. $Ca^{2+}$ and chloride parameters were taken from Ref.[7,8]. Equilibration simulations were carried out with GROMACS, version 2021[9]. Electrostatic interactions were described with the particle-mesh Ewald method[10,11]. Lennard-Jones interactions were truncated at 1.2 nm, while smoothly switching off the forces between 1.0 and 1.2 nm. The geometry of water molecules was constrained with the SETTLE algorithm[12]. The geometry of  bonds involving hydrogen atoms were constrained with p-LINCS[13]. To allow the use of a 4 fs integration time step, we used hydrogen mass repartitioning (HMR)[14], as implemented by the CHARMM-GUI service[15]. The temperature was controlled at 310 K using velocity rescaling, coupling membrane and solvent to separate heat baths ($\tau$ = 0.5 ps)[16]. The pressure was kept at 1 bar using the semi-isotropic Berendsen barostat, which we used owing to its numerical stability for equilibration simulations[17]. After energy minimization, the systems were equilibrated for 100 ns.

## S5. Free energy calculations of membrane pore formation

Potentials of mean force (PMFs) of pore formation were computed along the chain reaction coordinate $\xi_{ch}$, which quantifies the degree of connectivity of a polar defect over the lipid membrane [18,19]. The coordinate $\xi_{ch}$, is defined using a trans-membrane cylinder whose axis is aligned with the membrane normal (z direction) and whose center along z is is placed that membrane center of mass. The cylinder is decomposed into $N_s$ slices and $\xi_{ch}$ is defined as the fraction of slices that are filled by polar heavy atoms (here, oxygen atoms from water and lipid

phosphate groups). Hence, by pulling the simulation system along $\xi_{ch}$, the slices are filled with polar atoms one-by-one, thereby forming a continuous transmembrane polar defect. Harmonic restraints along $\xi_{ch}$, have been implemented into an in-house modification of GROMACS 2021, which is freely available at https://gitlab.com/cbjh/gromacs-chain-coordinate. For technical details we refer to Ref. [18]. Critically, in contrast to PMF calculations along several other reaction, PMF calculations along $\xi_{ch}$ do not suffer from hysteresis problems and free energy barriers are not integrated out[20].

An initial pore was formed using constant-velocity pulling from $\xi_{ch}$ = 0.1 to $\xi_{ch}$ = 1 over 100 ns with a force constant of 3000 kJ/mol along $\xi_{ch}$. To define $\xi_{ch}$, a cylinder with radius $R_{cyl}$ = 0.9 nm was used, and the cylinder was decomposed into slices with a thickness of $d_s$ = 0.1 nm. The number of slices $N_s$, was chosen such that 20% of the slices are filled by polar atoms ($\xi_{ch} \approx 0.2$) in the unperturbed membrane. This convention led to 26, 32, and 28 slices for the membranes of DOPC, DOPC:Chol and DPhPC, respectively. Fig. S2 shows the computed PMFs of pore formation for membranes of DOPC, DOPC:Chol 80:20, and DPhPC while applying transmembrane voltages of (A) 0 mV, (B) 300 mV, and (C) 600 mV as well as free energy of pore formation for all three cases.

The PMFs were computed with umbrella sampling using 27 umbrella windows. Initial frames for umbrella sampling were taken from the constant-velocity pulling simulation. We used the following non-equidistant spacing for the umbrella reference positions: 0.065 through 0.625 in steps of 0.08, and 0.64 through 1.0 in steps of 0.02. For reference positions smaller or larger than 0.7 we used force constants of 5000 or 10000 kJ/mol, respectively. Each window was simulated for 100 ns, where the first 20 ns were omitted for equilibration. PMFs were computed with the weighted histogram analysis method (WHAM), as implemented in the gmx wham module of GROMACS[21,22]. Statistical errors were estimated using 50 rounds of bootstrapping of complete histograms[22].

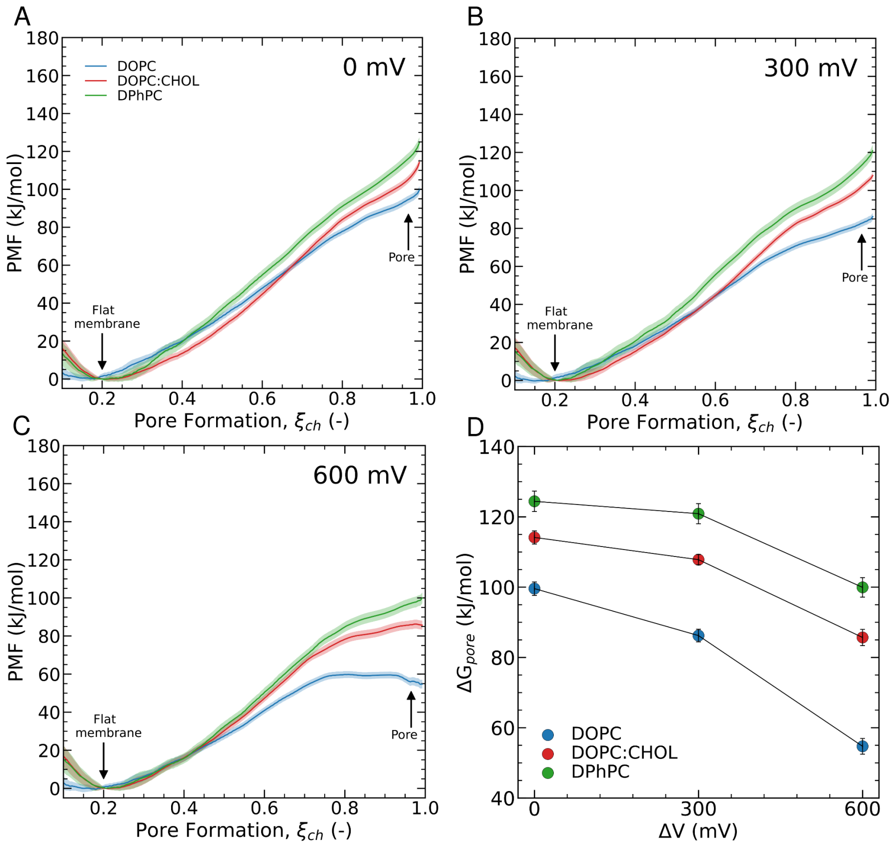


**Fig. S2.** PMFs of pore formation for membranes of DOPC, DOPC:Chol 80:20, and DPhPC (see legend for color code) while applying transmembrane voltages of (A) 0 mV, (B) 300 mV, and (C) 600 mV. (D) Free energy of pore formation versus transmembrane potential ΔV for the three lipid compositions.

## S6. Simulations of calcium permeation across an open pore

Systems for the simulation of $Ca^{2+}$ permeation across an open pore were set up with MemGen, with membrane patches composed of 162 DOPC lipids, 162 DPhPC lipids, 130 DOPC:32 Chol lipids, and 40 water molecules per lipid[2]. Water molecules were replaced with $Ca^{2+}$ and $Cl^{-}$ ions to obtain a concentration 1000 mM of $CaCl_2$. Hence, to obtain good permeation number statistics, we simulated a $CaCl_2$ concentration that is by far larger than physiological concentrations. The simulations were carried out with the Charmm36 lipid force field with electronic continuum correction (ECC), a Charmm36 variant with improved lipid–ion interactions[3–5]. $Ca^{2+}$ and chloride parameters were taken from Ref.[7,8]. The temperature was controlled at 310 K using velocity rescaling, coupling membrane and solvent to separate heat baths (τ = 1 ps)[16]. The pressure was kept at 1 bar using the semi-isotropic Parrinello-Rahman barostat[23]. No HMR was applied, and an integration of 2 fs was used. Other parameters were

used as described above. The membrane patches were equilibrated for 500 ns without any restraints.

A pore was induced in the equilibrated membranes by pulling along $\xi_{ch}$ from 0.1 to 1 over 100 ns using a force constant of 3000 kJ/mol. Then, the system was restrained at $\xi_{ch}$ = 1 with a force constant of 10000 and simulated for 500 ns. Fig. S3 shows the simulation snapshots of membranes with an open pore for DOPC, DOPC:Chol, and DPhPC lipids. The $Ca^{2+}$ permeation was obtained by counting the number of permeation events across the pore (in any direction, Fig. S4).

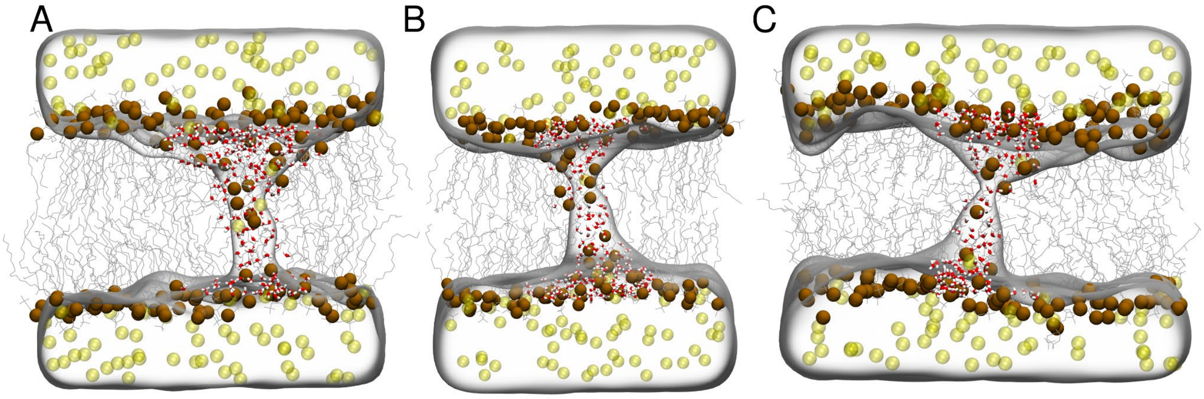


**Fig. S3.** Simulation snapshots of membranes with an open pore, maintained by restraining the systems to $\xi_{ch}$ = 1, for (A) DOPC, (B) DOPC:Chol 80:20, and (C) DPhPC. $Ca^{2+}$ ions are rendered as yellow spheres, lipid phosphorus atoms as brown spheres and water molecules inside the membrane as sticks. Water is in addition rendered as a transparent surface.

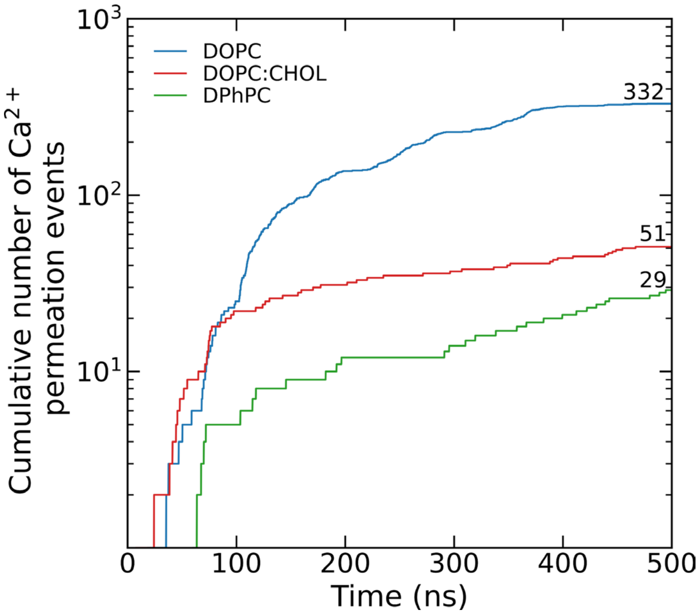


**Fig. S4.** Number of cumulative calcium permeation events versus simulation time for membranes at 600 mV with an open pore, maintained by restraining the systems to $\xi_{ch}$ = 1. The increased number of permeation events for DOPC as compared to DOPC:Chol or DPhPC is rationalized by the larger pore across the DOPC membrane.

## S7. 2PF imaging of $Ca^{2+}$ permeation

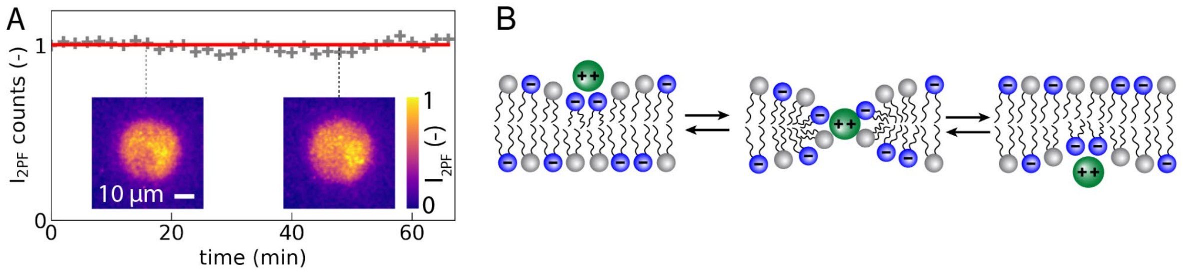


**Fig. S5: $Ca^{2+}$ permeation imaged by fluorescent probes.** (A) Fluorescence signal produced by Fluo-4 inside the DOPC:DOPA 1:1 GUV over time suggesting that $Ca^{2+}$ ions do not penetrate model lipid membranes. Inset: fluorescence images of a GUV with a Fluo-4 probe inside taken at different times after adding $CaCl_2$ to the outside solution. (B) Schematic illustration of a $Ca^{2+}$ ion passing through the membrane and remaining at the inner interface.

## References


1. Weinberger, A. *et al.* Gel-assisted formation of giant unilamellar vesicles. *Biophys. J.* **105**, 154–164 (2013).
2. Knight, C. J. & Hub, J. S. MemGen: a general web server for the setup of lipid membrane simulation systems. *Bioinformatics* **31**, 2897–2899 (2015).
3. Pastor, R. W. & MacKerell, A. D. Development of the CHARMM Force Field for Lipids. *J. Phys. Chem. Lett.* **2**, 1526–1532 (2011).
4. Melcr, J. *et al.* Accurate Binding of Sodium and Calcium to a POPC Bilayer by Effective Inclusion of Electronic Polarization. *J. Phys. Chem. B* **122**, 4546–4557 (2018).
5. Nencini, R. *et al.* Prosecco: polarization reintroduced by optimal scaling of electronic continuum correction origin in MD simulations. Available at: https://gitlab.com/sparkly/prosecco/prosECCo75. Accessed Oct 19, 2022.
6. Jorgensen, W. L., Chandrasekhar, J., Madura, J. D., Impey, R. W. & Klein, M. L. Comparison of simple potential functions for simulating liquid water. *J. Chem. Phys.* **79**, 926–935 (1983).
7. Kohagen, M., Mason, P. E. & Jungwirth, P. Accurate Description of Calcium Solvation in Concentrated Aqueous Solutions. *J. Phys. Chem. B* **118**, 7902–7909 (2014).
8. Kohagen, M., Mason, P. E. & Jungwirth, P. Accounting for Electronic Polarization Effects in Aqueous Sodium Chloride via Molecular Dynamics Aided by Neutron Scattering. *J. Phys. Chem. B* **120**, 1454–1460 (2016).
9. Abraham, M. J. *et al.* GROMACS: High performance molecular simulations through multi-level parallelism from laptops to supercomputers. *SoftwareX* **1–2**, 19–25 (2015).
10. Darden, T., York, D. & Pedersen, L. Particle mesh Ewald: An N·log(N) method for Ewald sums in large systems. *J. Chem. Phys.* **98**, 10089–10092 (1993).
11. Essmann, U. *et al.* A smooth particle mesh Ewald method. *J. Chem. Phys.* **103**, 8577–8593 (1995).
12. Miyamoto, S. & Kollman, P. A. Settle: An analytical version of the SHAKE and RATTLE algorithm for rigid water models. *J. Comput. Chem.* **13**, 952–962 (1992).

13. Hess, B. P-LINCS: A Parallel Linear Constraint Solver for Molecular Simulation. *J. Chem. Theory Comput.* **4**, 116–122 (2008).
14. Feenstra, K. A., Hess, B. & Berendsen, H. J. C. Improving efficiency of large time-scale molecular dynamics simulations of hydrogen-rich systems. *J. Comput. Chem.* **20**, 786–798 (1999).
15. Gao, Y. *et al.* CHARMM-GUI Supports Hydrogen Mass Repartitioning and Different Protonation States of Phosphates in Lipopolysaccharides. *J. Chem. Inf. Model.* **61**, 831–839 (2021).
16. Bussi, G., Donadio, D. & Parrinello, M. Canonical sampling through velocity rescaling. *J. Chem. Phys.* **126**, 014101 (2007).
17. Berendsen, H. J. C., Postma, J. P. M., van Gunsteren, W. F., DiNola, A. & Haak, J. R. Molecular dynamics with coupling to an external bath. *J. Chem. Phys.* **81**, 3684–3690 (1984).
18. Hub, J. S. & Awasthi, N. Probing a Continuous Polar Defect: A Reaction Coordinate for Pore Formation in Lipid Membranes. *J. Chem. Theory Comput.* **13**, 2352–2366 (2017).
19. Ting, C. L., Awasthi, N., Müller, M. & Hub, J. S. Metastable Prepores in Tension-Free Lipid Bilayers. *Phys. Rev. Lett.* **120**, 128103 (2018).
20. Awasthi, N. & Hub, J. S. Simulations of Pore Formation in Lipid Membranes: Reaction Coordinates, Convergence, Hysteresis, and Finite-Size Effects. *J. Chem. Theory Comput.* **12**, 3261–3269 (2016).
21. Kumar, S., Rosenberg, J. M., Bouzida, D., Swendsen, R. H. & Kollman, P. A. THE weighted histogram analysis method for free-energy calculations on biomolecules. I. The method. *J. Comput. Chem.* **13**, 1011–1021 (1992).
22. Hub, J. S., de Groot, B. L. & van der Spoel, D. g_wham—A Free Weighted Histogram Analysis Implementation Including Robust Error and Autocorrelation Estimates. *J. Chem. Theory Comput.* **6**, 3713–3720 (2010).
23. Parrinello, M. & Rahman, A. Polymorphic transitions in single crystals: A new molecular dynamics method. *J. Appl. Phys.* **52**, 7182–7190 (1981).
